# MRI-based radiomics in breast cancer: feature robustness with respect to inter-observer segmentation variability

**DOI:** 10.1038/s41598-020-70940-z

**Published:** 2020-08-25

**Authors:** R. W. Y. Granzier, N. M. H. Verbakel, A. Ibrahim, J. E. van Timmeren, T. J. A. van Nijnatten, R. T. H. Leijenaar, M. B. I. Lobbes, M. L. Smidt, H. C. Woodruff

**Affiliations:** 1 Department of Surgery, Maastricht University Medical Center+, P.O. Box 5800, 6202 AZ Maastricht, The Netherlands; 2 GROW – School for Oncology and Developmental Biology, Maastricht University, Maastricht, The Netherlands; 3 Department of Radiology and Nuclear Medicine, Maastricht University Medical Center+, Maastricht, The Netherlands; 4 The D-Lab, Department of Precision Medicine, Maastricht University, Maastricht, The Netherlands; 5 Division of Nuclear Medicine and Oncological Imaging, Department of Medical Physics, Hospital Center Universitaire De Liege, Liège, Belgium; 6 Department of Nuclear Medicine and Comprehensive Diagnostic Center Aachen (CDCA), University Hospital RWTH Aachen University, Aachen, Germany; 7 Department of Medical Imaging, Zuyderland Medical Center, Sittard-Geleen, The Netherlands

**Keywords:** High-throughput screening, Data acquisition, Data processing, Breast cancer

## Abstract

Radiomics is an emerging field using the extraction of quantitative features from medical images for tissue characterization. While MRI-based radiomics is still at an early stage, it showed some promising results in studies focusing on breast cancer patients in improving diagnoses and therapy response assessment. Nevertheless, the use of radiomics raises a number of issues regarding feature quantification and robustness. Therefore, our study aim was to determine the robustness of radiomics features extracted by two commonly used radiomics software with respect to variability in manual breast tumor segmentation on MRI. A total of 129 histologically confirmed breast tumors were segmented manually in three dimensions on the first post-contrast T1-weighted MR exam by four observers: a dedicated breast radiologist, a resident, a Ph.D. candidate, and a medical student. Robust features were assessed using the intraclass correlation coefficient (ICC > 0.9). The inter-observer variability was evaluated by the volumetric Dice Similarity Coefficient (DSC). The mean DSC for all tumors was 0.81 (range 0.19–0.96), indicating a good spatial overlap of the segmentations based on observers of varying expertise. In total, 41.6% (552/1328) and 32.8% (273/833) of all RadiomiX and Pyradiomics features, respectively, were identified as robust and were independent of inter-observer manual segmentation variability.

## Introduction

Radiomics is a technique that is used to extract large amounts of quantitative information from routine medical images that decode information about a region of interest (ROI). The majority of radiomics articles published concerns its application in the oncological field^1–4^. Here, radiomics bears the advantage of non-invasively quantifying the underlying phenotype of the entire tumor for multiple lesions simultaneously, in contrast to tissue biopsy, which samples only a small part of a single (often heterogeneous) tumor^2,5^. The ability to characterize the tumor and to establish links to the underlying biology^6^ and ultimately clinical outcomes, allows a more patient-tailored treatment^7^, enabling ‘precision medicine’^8,9^. Recently, several articles have outlined the potential clinical applicability of radiomics in the field of breast cancer for different purposes, e.g. diagnosis^10,11^, tumor response prediction^12–14^, prediction of molecular tumor subtype^15,16^, and prediction of axillary lymph node metastases^17,18^.

Although these results are promising, issues regarding features robustness as well as the comparability of results, including inter-observer segmentation variability, need to be addressed^19–24^. In order to extract clinically useful information from medical images and to use features as clinical biomarkers, it is important that extracted features are reproducible, standardized and robust^25,26^. All consecutive steps in the radiomics workflow induce potential uncertainties regarding feature robustness^27,28^. Since there used to be no gold standard or guideline for extraction of image features for radiomics use, an initiative –Image Biomarker Standardization Initiative (IBSI)- was launched as an effort to standardize the entire radiomics extraction process and encourage feature robustness^29^.

ROI segmentation is an important step after image acquisition in the radiomics workflow, and one of the largest bottlenecks^30^. Traditionally, the edges (2D) or surfaces (3D) of the ROI are segmented, thereby defining a region from which features will be extracted. Segmentation can be performed either manually, semi-automatically, or completely automatically. Both manual and semi-automatic segmentation are prone to inter- and intra-observer variabilities, with the degree of observer experience playing an important role^31–33^.

To the best of our knowledge, no articles have been published on the effect of manual inter-observer segmentation variability on MRI-based feature robustness in breast cancer patients. MRI is the most accurate modality for neoadjuvant systemic therapy response monitoring in breast cancer patients and as such much used in daily clinical practice^34–37^. In this article, we investigate the robustness of MR radiomics features, extracted using two commonly used radiomics software, with respect to variations in manual tumor segmentation of breast cancer patients.

## Results

### Study population

After the application of inclusion and exclusion criteria, 102 patients were included in the final analysis. Twenty-one of these patients were diagnosed with multifocal breast cancer, bringing the total number of tumors analyzed in this study to 129. Of these, 94 tumors (73%) were assigned ‘easy tumors’ and the remaining 35 tumors (27%) were assigned ‘challenging tumors’. The tumor volume between both groups was significant differently (5.3 vs 10.4 for ‘easy and challenging tumors’, respectively, *p* = 0.03).

### Segmentation variability

**Figure 1:**
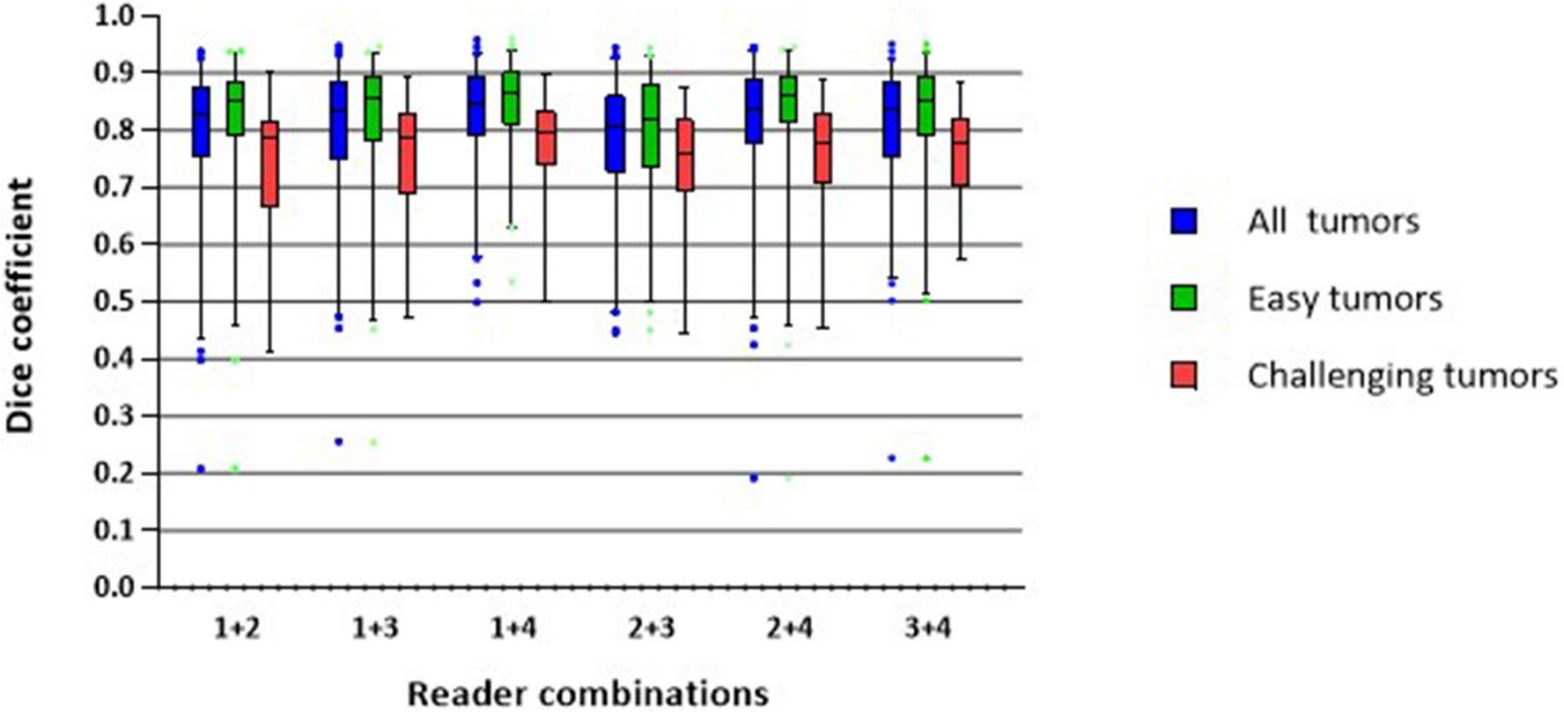
Tumor segmentation variability for pairwise comparison of the different observers. (1) Dedicated breast radiologist, (2) Radiology resident, (3) Ph.D. candidate with a medical degree and (4) Medical student.

DSC distributions of all observer combinations are shown in Fig. 1. The mean DSC was 0.81 (range 0.19–0.96). The mean DSC was higher for the ‘easy tumors’ compared to the ‘challenging tumors’ (0.83 vs. 0.75, respectively, *p* < 0.001). The mean DSC for each observer combination separately, for all tumors, ranged between 0.78 and 0.83, where the segmentations of the breast radiologist and the medical student showed the highest overlap.

### Pre-processing and feature extraction

The bin width for image discretization (calculated from the ROI greyscale range) was 0.1. Discretization of the scans with bins 0.1 wide resulted in a mean of 61 grayscale values per image (range 27–131). RadiomiX and Pyradiomics software extracted a total of 1328 and 833 features for each ROI, respectively. The extracted radiomics features included shape features, first-order statistical, intensity-histogram based, fractal, local intensity, and texture matrix-based features from both unfiltered and filtered images (wavelet decompositions). The RadiomiX software extracts more feature groups compared to the Pyradiomics software, namely intensity histogram (IH), fractal, local intensity, and gray level dependency zone matrix (GLDZM) features.

### Radiomics feature robustness

The average ICC for all RadiomiX features was 0.86 (95% CI 0.85–0.86) and for all Pyradiomics features 0.84 (95% CI 0.83–0.84). Table 1 presents the average ICC value per feature group for both software. The local intensity features scored the highest average ICC value for the RadiomiX features, and the first-order statistical features score the highest average ICC for the Pyradiomics features.

**Table 1:** Average ICC values per feature group of the unfiltered and wavelet RadiomiX and Pyradiomics features.

Feature group (n)	OncoRadiomiX		Pyradiomics	
	Mean ICC	Range	Mean ICC	Range
Shape	0.79	0.57–0.93	0.80	0.69–0.92
**Signal intensity**				
First-order statistics	0.85	0.51–0.99	0.84	0.50–0.97
IH	0.76	0.63–0.98	–	–
Fractal	0.81	0.79–0.83	–	–
LocInt	0.95	0.93–0.96	–	–
GLCM	0.76	0.49–0.88	0.80	0.71–0.88
GLRLM	0.79	0.56–0.96	0.81	0.63–0.95
GLSZM	0.80	0.55–0.98	0.84	0.58–0.97
GLDZM	0.76	0.50–0.92	–	–
NGTDM	0.78	0.57–0.85	0.80	0.72–0.91
(N)GLDM	0.83	0.55–0.96	0.79	0.52–0.96
Wavelet	0.81	0.01–0.99	0.81	0.12–0.99

**Figure 2:**
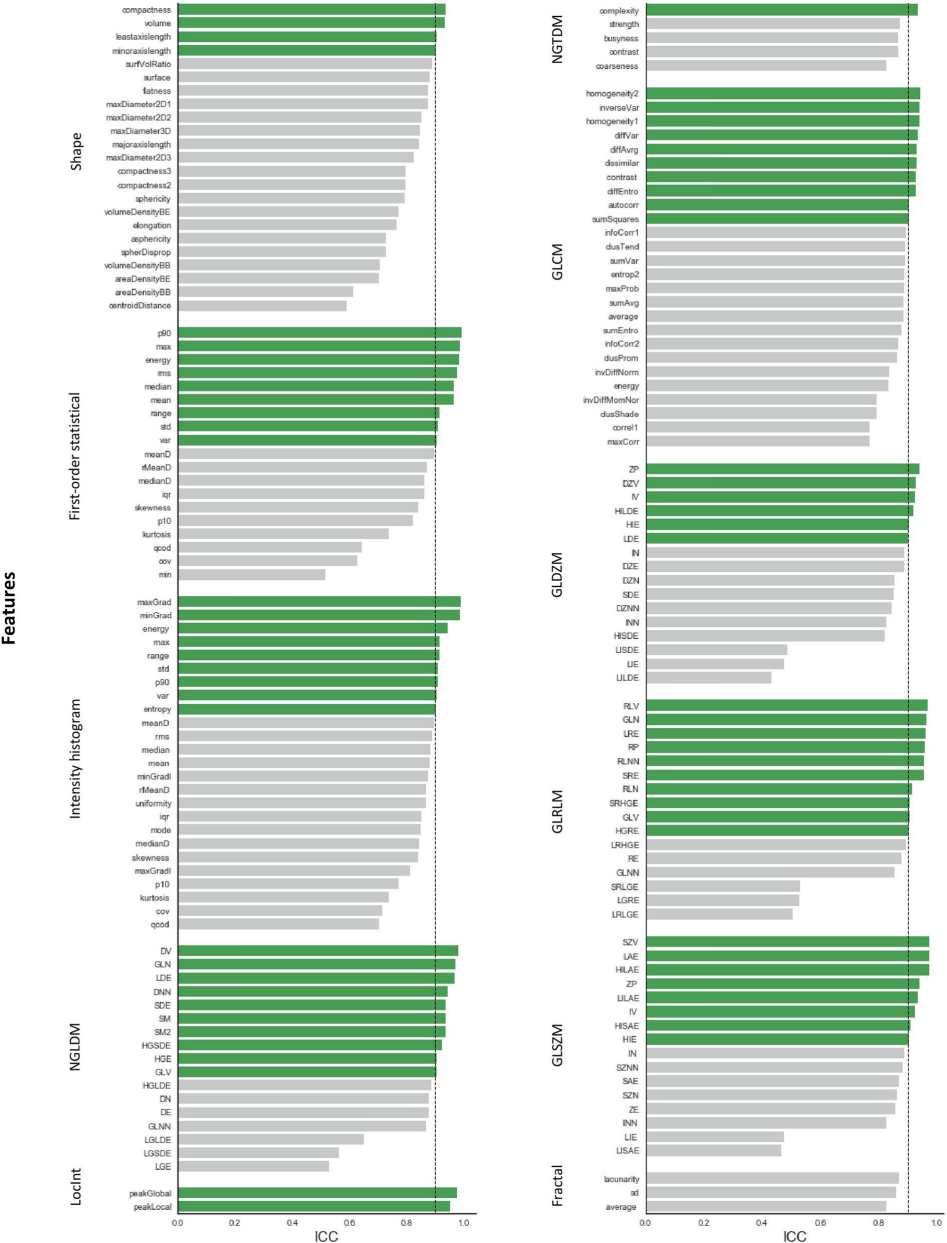
ICC values of all unfiltered RadiomiX features with robust features (ICC > 0.90) shown in green.

**Figure 3:**
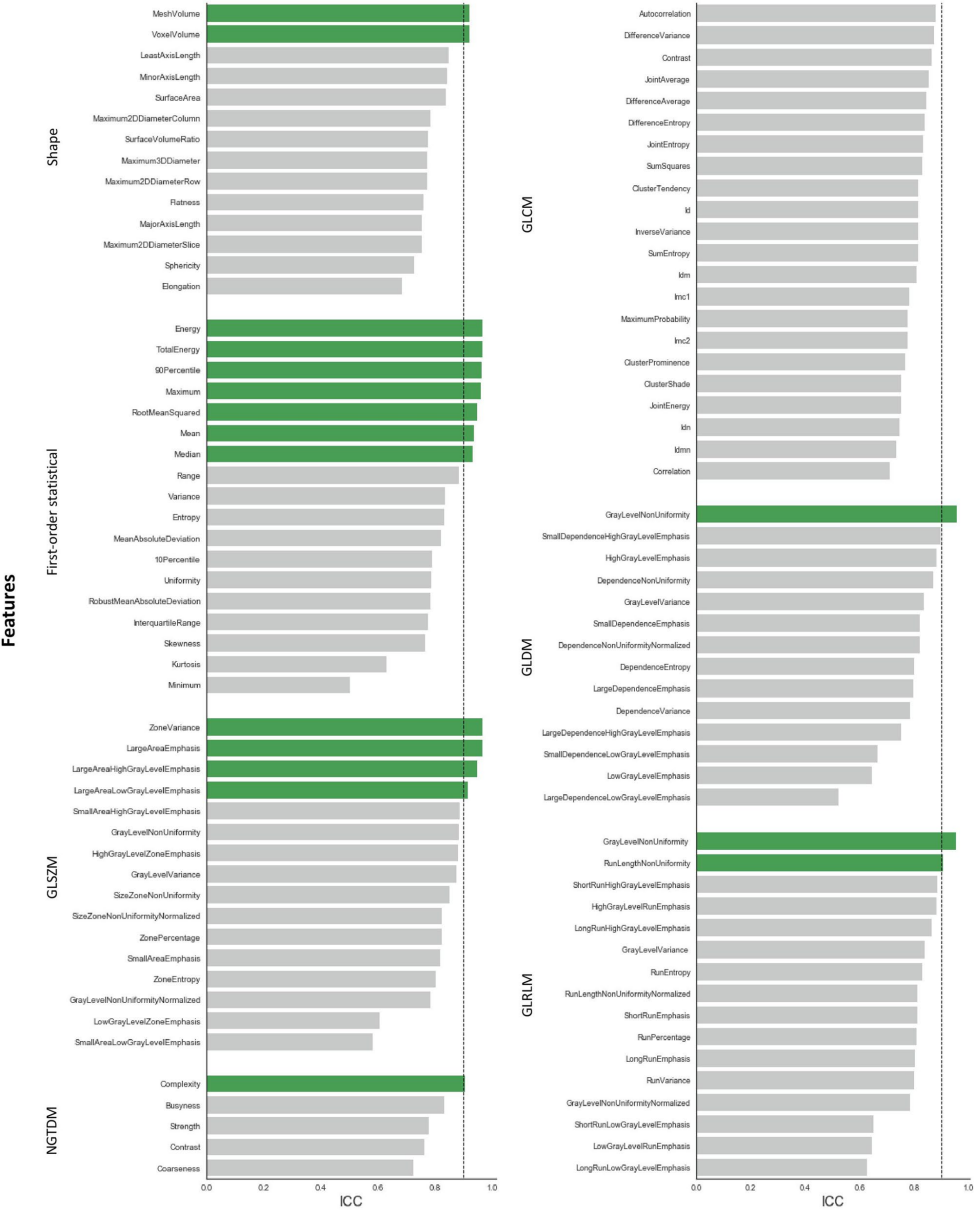
ICC values of all unfiltered Pyradiomics features with robust features (ICC > 0.90) shown in green.

The percentage of features that scored an ICC > 0.90, and thus were labeled by our pre-determined ICC cut-off as robust, was 41.6% (552/1328) for RadiomiX features and 32.8% (273/833) for Pyradiomics features. The unfiltered RadiomiX features (i.e., calculated on the unfiltered images) had an average ICC value of 0.79 (95% CI 0.77–0.81), of which 41.1% (69/168) were robust (Fig. 2). The unfiltered Pyradiomics features had an average ICC value of 0.81 (95% CI 0.79–0.83), of which 16.2% (17/105) were robust (Fig. 3). The results of the wavelet feature groups for both software are presented in the supplementary material 1 and 2.

The percentage of robust RadiomiX features for the ‘easy tumors’ and the ‘challenging tumors’ was 57.5% (763/1328) and 17.2% (228/1328), respectively. When only considering the 168 unfiltered features, 50.0% (84/168) of the ‘easy tumors’ were robust and 20.2% (34/168) of the ‘challenging tumors’ (supplementary material 3). The percentage of robust Pyradiomics features for the ‘easy tumors’ and the ‘challenging tumors’ was 35.7% (297/833) and 28.6% (238/833), respectively. When only considering the 105 unfiltered features, 23.8% (25/105) of the ‘easy tumors’ were robust and 14.3% (15/105) of the ‘challenging tumors’ (supplementary material 4).

## Discussion

In this study, our ultimate goal was to define a list of robust MRI radiomics features, independent of inter-observer segmentation variability, which could facilitate further breast MRI-based radiomics research. We successfully identified a subgroup of robust features for two commonly used radiomics software (41.6% of all RadiomiX features and 32.8% of all Pyradiomics features) in the presence of inter-observer segmentation variability (mean DSC of 0.81).

Although MRI feature robustness has already been investigated for different tumor sites (*e.g.,* cervical cancer^19^ and glioblastoma^23^), the effect of inter-observer variability segmentation is most likely tumor-site specific^38^. The feature groups enclosing the most robust features in previous investigations (shape^19^ and, Intensity-histogram and GLCM^23^) are different from what we found to be the feature group enclosing the most robust features (local intensities and GLRLM). Most likely this could be explained that different tumor sites influence inter-observer variability. Although one must not forget that the differences in MRI sequences and, feature extraction software also influence this variability. Therefore, the MRI feature robustness cannot be generalized and must be examined for each specific tumor site, taking into account different MRI sequences and feature extraction software.

In addition, feature robustness for both radiomics software was identified for ‘easy tumors’ and ‘challenging tumors’. The number of robust features increased for ‘easy tumors’ and decreased for ‘challenging tumors’ in both software with significant differences between the mean DSC of the ‘easy’ and ‘challenging’ tumors (0.83 vs. 0.75, respectively, *p* < 0.001). The fact that the ‘challenging tumors’ were more irregular, often with spiculae, causes more segmentation variability and therefore less robust features. Furthermore, the significant difference in the DSC between easy and challenging tumors could be attributed to the sensitivity of the metric to tumor volume. Easy tumors were on average significantly smaller than challenging ones; therefore, a minor difference in segmentation of a small tumor would have a more profound effect on the DSC, compared to those with larger volumes.

A detailed comparison to previous studies is limited to one similar study. Saha et al.^39^ investigated the impact of breast MRI segmentation variability on radiomics feature robustness, whereby features were extracted using in-house software. Their reported mean ICC of 0.85 for all features, using semi-automatic breast tumor segmentation, is comparable to the average ICC reported in this study. Although the segmentations were performed by four fellow breast radiology trainees, the DSC results they report (range 0.506–0.740) were much lower than the DSC results in our analysis (range 0.783–0.827). We consciously opted for people with different segmentation expertise to ensure observer-independence of the robust features, consequently widening the applicability. Approximately 10% of the tumor features in their article were found to be robust, compared to 41.1% in this study. Solely 20 textural features (GLCM) were comparable between the studies, whereby the ICC of these features showed a substantial difference (average 0.26, range 0.09–0.51).

While we present the robust features for two different radiomics software, our aim is solely to facilitate future application of our findings. Both software have different pre-processing steps, and different groups of features, and comparing the software is beyond the scope of this study. A global initiative to standardize radiomic features extraction using different radiomics software–Imaging Biomarkers Standardization Initiative (IBSI)- was started to address these issues in a more comprehensive fashion^40^.

To overcome the problem of inter-observer variability with respect to ROI segmentation, promising steps towards (semi-)automatic segmentation have been taken in other tumor sites^41–45^. However, little work has been published on fully automatic segmentation software for DCE-MRI of the breast^33,46–48^. Most software, including semi-automatic segmentation, still require manual input or adjustments^33,46,47^, and would still be significantly slower than fully automated segmentation. Recent work on automatic MRI breast tissue segmentation reported encouraging results but was performed on only 30 patients^48^. The current lack of reliable, validated and widely available automatic segmentation software tools, and the need for manual input in semi-automated segmentation, demonstrate that manual segmentation remains important. The use of protocols or guidelines could encourage more reproducible manual segmentation results^49,50^. Furthermore, by providing precise instructions before the start of segmentation, inter-observer segmentation variability can be minimized.

There are some limitations to this study. Although an ICC threshold value of 0.90 was chosen to determine feature robustness, the significance of this threshold for radiomics models for patients’ outcome prediction is yet to be investigated. The inclusion of more patients and observers will allow better generalization of the results and development of robust radiomics signatures. Furthermore, we identified feature robustness to segmentation observer variability. However, due to the lack of data, we were not able to assess the robustness of radiomics features to differences in image acquisition, pre-processing and feature extraction, which are other major challenges in radiomics analysis. These are the aim of our current studies.

In conclusion, this study shows the intuitive notion that more complex, challenging tumors lead to less robust features. We identified radiomics features robust to inter-observer variations across two different radiomics software, which could be used for preselection of radiomics features in future radiomics analysis concerning MRI-based breast radiomics. Ultimately, this study identified a list of robust radiomics features, which is independent of inter-observer segmentation variability in breast MRI for two commonly used software.

## Material and methods

### Study population

**Figure 4:**
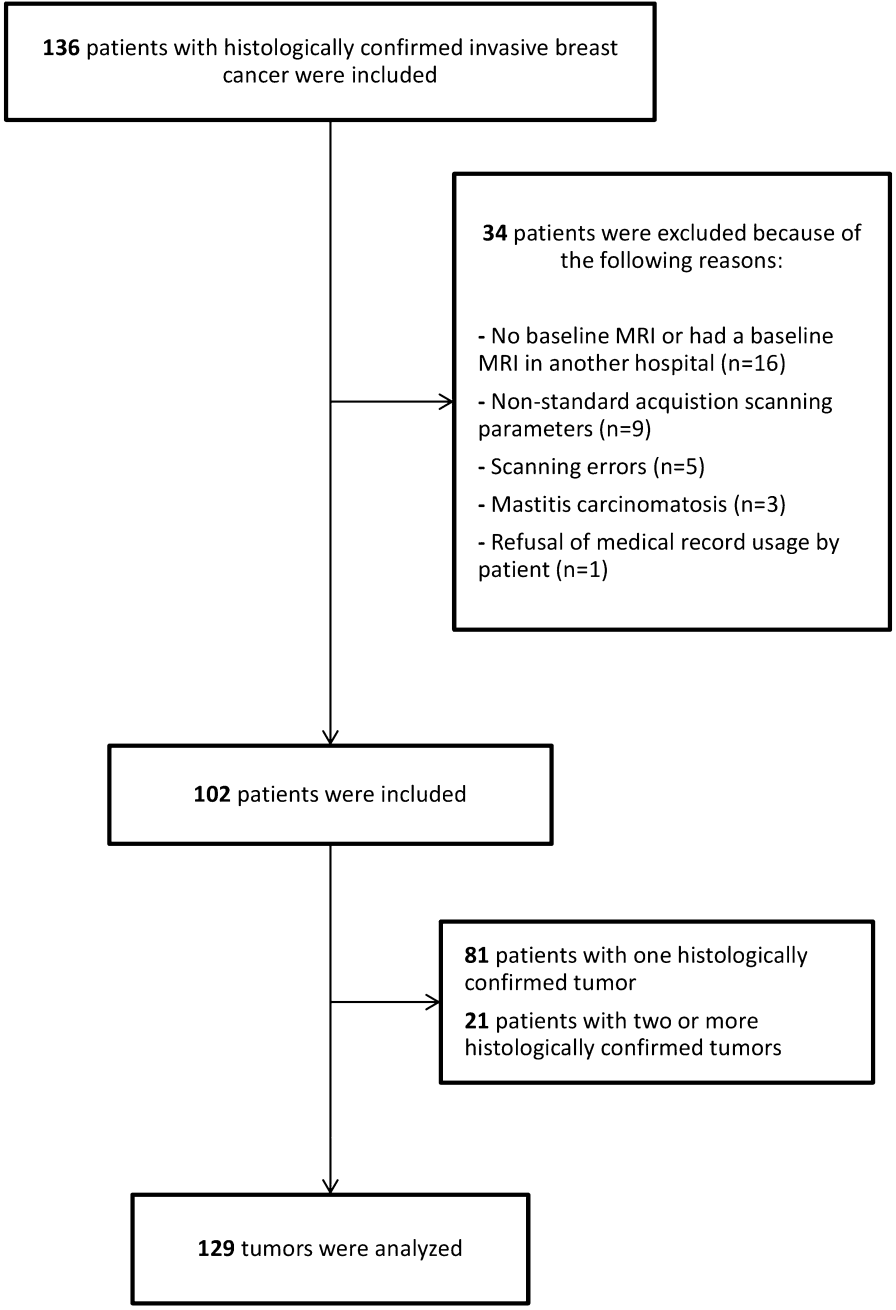
Flowchart of the patient population in the study.

In this single-center retrospective study, we collected data on 138 patients with histologically confirmed invasive breast cancer, who were planned for receiving NST and underwent a pretreatment DCE-MRI between January 2011 and December 2017 in Maastricht University Medical Center+. The institutional research board of the MUMC+ approved the study and waived the requirement for informed consent and the further need of guidelines. Exclusion criteria were: pathologically confirmed mastitis carcinomatosa, MR scan artifacts, or refusal of medical record usage by the patient. Furthermore, we excluded patients that underwent breast MRI exams with non-standard acquisition parameters, due to the use of a different MRI scanner. All histologically confirmed breast tumors were included in the analysis. The complete process is summarized in the flowchart presented in Fig. 4.

### Imaging data

All images were acquired by two clinically interchangeable (i.e. provide qualitatively similar images) 1.5 T MRI scanners (Philips Intera and Philips Ingenia), using a dynamic contrast-enhanced T1-weighted (DCE-T1W) sequence with similar acquisition protocols (Table 2). The patients were scanned in prone position using a 16-channel dedicated breast coil. The DCE-T1W images were obtained before and after intravenous injection of gadolinium-based contrast Gadobutrol (Gadovist (EU)) with a volume of 15 cc and a flow rate of 2 ml/s. One pre-contrast image and five post-contrast images were obtained for each patient.

**Table 2:** Imaging parameters for the breast DCE T1W sequence for both scanners.

	Scanner 1 Philips Ingenia (n)	Scanner 2 Philips Intera (n)
Number of tumors	100	29
Field strength (T)	1.5	1.5
Slice thickness (mm)	1.0	1.0
Repetition time (ms)	7.5 (88), 7.6 (12)	7.4 (13), 7.5 (15), 7.6 (1)
Echo time (ms)	3.4	3.4
Flip angle (°)	10	10
Echo train length	89* (range 62–175)	80* (range 60–85)
Pixel spacing (mm)	0.79^2^ (3), 0.85^2^ (1), 0.92^2^ (2), 0.95^2^ (47), 0.95^2^ (47)	0.85^2^ (1), 0.94^2^ (1), 0.97^2^ (26), 0.99^2^ (1)
Temporal resolution (s)	95	98

*Average.

### Tumor segmentation

**Figure 5:**
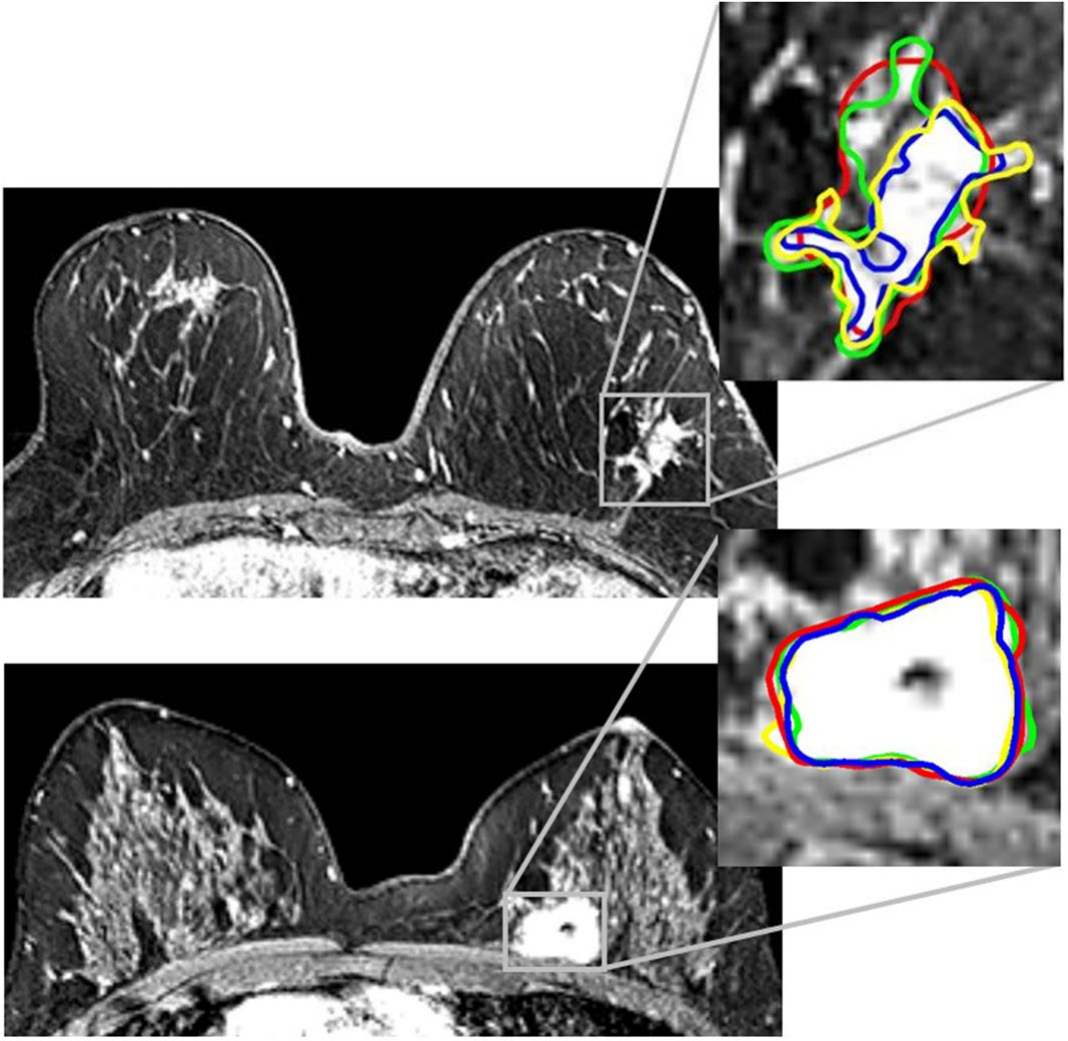
Two invasive breast tumors in the left breast on the 2-min post-contrast DCE-MRI with four single manual segmentations (colored margins: red, blue, green and yellow) fused. Upper: ‘challenging tumor’ with a mean DSC of 0.78 (range 0.71–0.82). Lower: ‘easy tumor’ with a mean DSC of 0.90 (range 0.89*–*0.91).

The T1W images acquired two minutes post-contrast administration were used for the 3D tumor segmentation, as this is generally accepted to be the peak of enhancement of breast cancers^51^. Tumors were independently segmented by four observers with different degrees of experience in breast MR imaging: a dedicated breast radiologist with 11 years of clinical breast MRI experience (ML), a radiology resident with one year of breast MRI clinical experience (TvN), a Ph.D. candidate with a medical degree but no breast MRI clinical experience (RG) and a medical student with no experience whatsoever (NV) (Fig. 5). Segmentations were performed manually with Mirada RTx (v1.2.0.59, Mirada Medical, Oxford, UK). Agreements regarding segmentation procedures were made prior to tumor segmentation: (i) observers were allowed to adjust the image grayscale to optimize the visualization of the tumor; (ii) lymph nodes, pectoral muscle, and skin were excluded from segmentation; (iii) spiculae were only segmented if histologically confirmed. All observers had access to the radiology report during segmentation but were blinded to each other’s segmentations.

### Image pre-processing and feature extraction

Radiomics feature extraction is generally performed after image pre-processing. Pre-processing is designed to increase data homogeneity, as well as to reduce image noise and computational requirements. Both radiomics software have the optionality to perform image normalization internally before feature extraction, which varies to an extent across the software. Pyradiomics centers the image around the mean and standard deviation based on all gray values of the image, while RadiomiX normalizes the images after removal of background data (non-breast voxels containing air). This transforms the voxel grayscale values to a more comparable range without changing image textures. Each image was discretized by resampling the grayscale values using a fixed bin width of 0.1 in order to reduce image noise and computational burden. The Pyradiomics community^52^ recommends the number of bins to be in range of 16–128. We calculated the optimal bin width by extracting the greyscale ranges within all the ROIs and choosing a width that maximizes the number of ROIs that fall in the abovementioned range of bins. Finally, voxel size was standardized across the cohorts to isotropic 1.0 mm^3^ voxels by means of linear interpolation. For each manually segmented ROI, features were extracted using two commonly used radiomics software: RadiomiX Discovery Toolbox software (OncoRadiomics SA, Liège, Belgium) and the open-source Pyradiomics software, version 2.1.2^52,53^. A mathematical description of all RadiomiX features can be found in supplementary material 5. The Pyradiomics feature description can be found online^54^. Both software are IBSI compliant for most features, with a note being added in case of differences.

### Data analyses

#### Segmentation variability analysis

Features with (near) zero variance across all tumors, i.e. features that have the same value across ninety-five percent or more of the observations, were excluded from the analysis as they carry no discriminative value. To evaluate the variability of the remaining features introduced by manual segmentation, the volumetric Dice Similarity Coefficient (DSC) was calculated for all pairs of observers. The DSC is a metric that quantifies the agreement (or ‘overlap’) between two segmentations^55^. A DSC of 1 indicates perfect spatial overlap of the segmentations, whereas 0 indicates no agreement, i.e. no spatial overlap of the segmentations, and a good overlap is considered with DSC > 0.7 as indicated by the literature^56^. The DSC was calculated as:

$${\text{DSC}} = 2\frac{{(\left| {A \cap B} \right|)}}{(\left| A \right| + \left| B \right|)}$$

where A is the set of voxels contained in the first contour, B is the set of voxels contained in the second contour, || indicates the cardinality of the sets, and ∩ is the intersection between the first and second sets^57^. The DSC was calculated using Python (Version 3.6.3150.1013).

#### Radiomics feature robustness analysis

Feature robustness was assessed by evaluating the two-way random single measure intraclass correlation coefficient (ICC) (2,1). The two-way random model approach was chosen as it allows generalization of the results to any other rater with similar characteristics^57^. The ICC ranges between 0 and 1, with values closer to 1 representing stronger feature robustness to differences in segmentations. We chose a pre-defined ICC cut-off of > 0.9 to select highly stable features that are insensitive to segmentation variability^57^. Feature robustness was calculated for all RadiomiX and Pyradiomics features. The settings for image pre-processing (normalization, discretization, and resampling) in both radiomics software were checked for disparities. Calculations were performed in R studio (version 1.1.456, Vienna, Austria)^58^ using the IRR package version 0.84^59^.

#### Easy- versus challenging-to-segment tumors analysis

The differences in feature robustness and inter-observer tumor segmentation variability between ‘easy-to-segment’ and ‘challenging-to-segment’ tumors ones, hereinafter referred to as ‘easy tumors’ and ‘challenging tumors’, were assessed. This classification was unanimously determined by the dedicated breast radiologist (ML). ‘Easy tumors’ were defined as homogenous, round tumors with relatively sharp (albeit sometimes irregular) margins, without spiculae or areas of accompanying non-mass enhancement. Tumors not meeting these criteria were categorized as ‘challenging tumors’ (Fig. 5). To compare DSC results between ‘easy’ and ‘challenging’ tumors we used the independent samples t-test, performed in R studio using the IRR package.

## Supplementary information

Supplementary Information 1.
