# Supplementary material for: MRI-based radiomics in breast cancer: feature robustness with respect to inter-observer segmentation variability
Source: Sci Rep. 2020 Aug 25;10:14163. doi: 10.1038/s41598-020-70940-z (PMC7447771; doi:10.1038/s41598-020-70940-z)
Supplement: Supplementary file 1 — Supplementary Information 1. [file 41598_2020_70940_MOESM1_ESM.pdf]

MRI-based radiomics in breast cancer: feature robustness with respect to inter-observer segmentation variability.

Supplementary material

Granzier RWY, MD[1,2], Verbakel NMH[1*], Ibrahim A, MD[2,3,4,5,6*], van Timmeren JE, PhD[2,4], van Nijnatten TJA, MD, PhD[3], Leijenaar RTH, PhD[2,4], Lobbes MBI, MD, PhD[2,3,7], Smidt ML, MD, PhD[1,2†], Woodruff HC, PhD[2,3,4†]

[*]Authors contributed equally
† Senior authors contributed equally

Author affiliations

[1] Department of Surgery, Maastricht University Medical Centre+, Maastricht, the Netherlands.

[2] GROW – School for Oncology and Developmental Biology, Maastricht University, Maastricht, the Netherlands.

[3] Department of Radiology and Nuclear Medicine, Maastricht University Medical Centre+, Maastricht, The Netherlands.

[4] The D-Lab, Department of Precision Medicine, Maastricht University, Maastricht, The Netherlands.

[5] Division of Nuclear Medicine and Oncological Imaging, Department of Medical Physics, Hospital Center Universitaire De Liege, Liege, Belgium.

[6] Department of Nuclear Medicine and Comprehensive diagnostic center Aachen (CDCA), University Hospital RWTH Aachen University, Aachen, Germany

[7] Department of Medical Imaging, Zuyderland Medical Center, Sittard-Geleen, the Netherlands.

Corresponding author

Renée Granzier, MD
Department of Surgery
Maastricht University Medical Center+
P.O. Box 5800
6202 AZ Maastricht
The Netherlands
Phone: +31 43 388 2354
Email address: r.granzier@maastrichtuniversity.nl

Supplementary material 1. ICC values of all wavelet RadiomiX features per wavelet decomposition with robust features (ICC > 0.9) shown in green

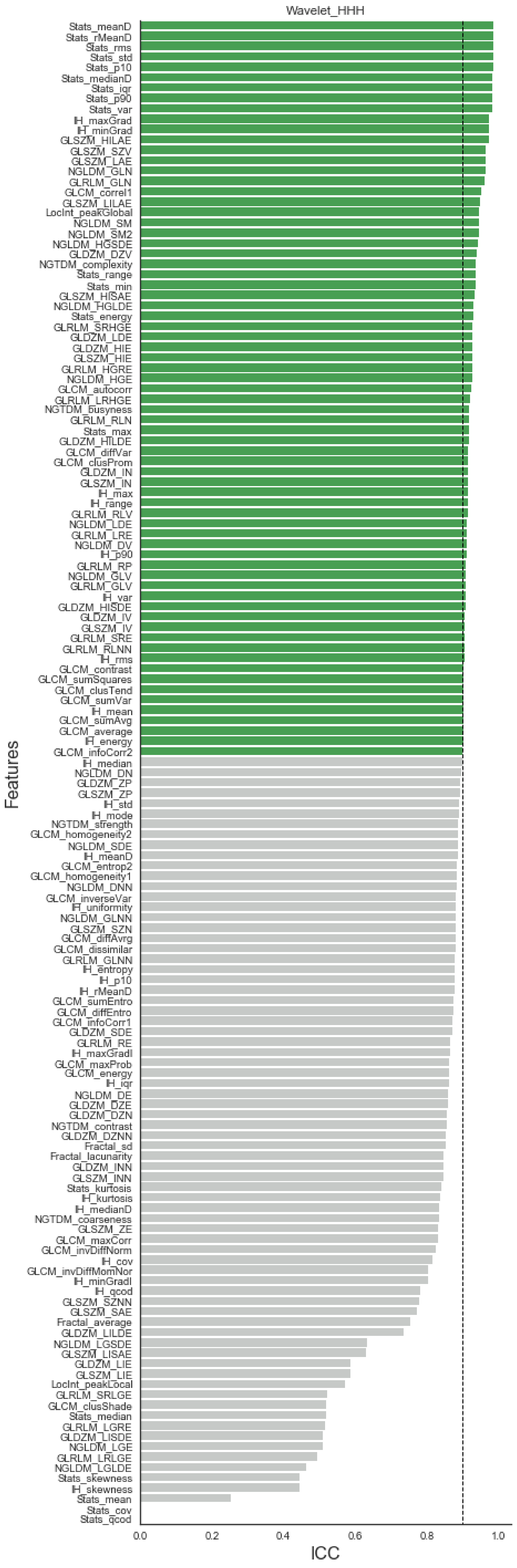
Wavelet_HHH
Stats_meanD
Stats_rMeanD
Stats_rms
Stats_std
Stats_p10
Stats_medianD
Stats_iqr
Stats_p90
Stats_var
IH_maxGrad
IH_minGrad
GLSZM_HILAE
GLSZM_SZV
GLSZM_LAE
NGLDM_GLN
GLRLM_GLN
GLCM_correl1
GLSZM_LILAE
LocInt_peakGlobal
NGLDM_SM
NGLDM_SM2
NGLDM_HGSDE
GLDZM_DZV
NGTDM_complexity
Stats_range
Stats_min
GLSZM_HISAE
NGLDM_HGLDE
Stats_energy
GLRLM_SRHGE
GLDZM_LDE
GLDZM_HIE
GLSZM_HIE
GLRLM_HGRE
NGLDM_HGE
GLCM_autocorr
GLRLM_LRHGE
NGTDM_busyness
GLRLM_RLN
Stats_max
GLDZM_HILDE
GLCM_diffVar
GLCM_clusProm
GLDZM_IN
GLSZM_IN
IH_max
IH_range
GLRLM_RLV
NGLDM_LDE
GLRLM_LRE
NGLDM_DV
IH_p90
GLRLM_RP
NGLDM_GLV
GLRLM_GLV
IH_var
GLDZM_HISDE
GLDZM_IV
GLSZM_IV
GLRLM_SRE
GLRLM_RLNN
IH_rms
GLCM_contrast
GLCM_sumSquares
GLCM_clusTend
GLCM_sumVar
IH_mean
GLCM_sumAvg
GLCM_average
IH_energy
GLCM_infoCorr2
IH_median
NGLDM_DN
GLDZM_ZP
GLSZM_ZP
IH_std
IH_mode
NGTDM_strength
GLCM_homogeneity2
NGLDM_SDE
IH_meanD
GLCM_entrop2
GLCM_homogeneity1
NGLDM_DNN
GLCM_inverseVar
IH_uniformity
NGLDM_GLNN
GLSZM_SZN
GLCM_diffAvrg
GLCM_dissimilar
GLRLM_GLNN
IH_entropy
IH_p10
IH_rMeanD
GLCM_sumEntro
GLCM_diffEntro
GLCM_infoCorr1
GLDZM_SDE
GLRLM_RE
IH_maxGradI
GLCM_maxProb
GLCM_energy
IH_iqr
NGLDM_DE
GLDZM_DZE
GLDZM_DZN
NGTDM_contrast
GLDZM_DZNN
Fractal_sd
Fractal_lacunarity
GLDZM_INN
GLSZM_INN
Stats_kurtosis
IH_kurtosis
IH_medianD
NGTDM_coarseness
GLSZM_ZE
GLCM_maxCorr
GLCM_invDiffNorm
IH_cov
GLCM_invDiffMomNor
IH_minGradI
IH_qcod
GLSZM_SZNN
GLSZM_SAE
Fractal_average
GLDZM_LILDE
NGLDM_LGSDE
GLSZM_LISAE
GLDZM_LIE
GLSZM_LIE
LocInt_peakLocal
GLRLM_SRLGE
GLCM_clusShade
Stats_median
GLRLM_LGRE
GLDZM_LISDE
NGLDM_LGE
GLRLM_LRLGE
NGLDM_LGLDE
Stats_skewness
IH_skewness
Stats_mean
Stats_cov
Stats_qcod
Features
0.0
0.2
0.4
0.6
0.8
1.0
ICC

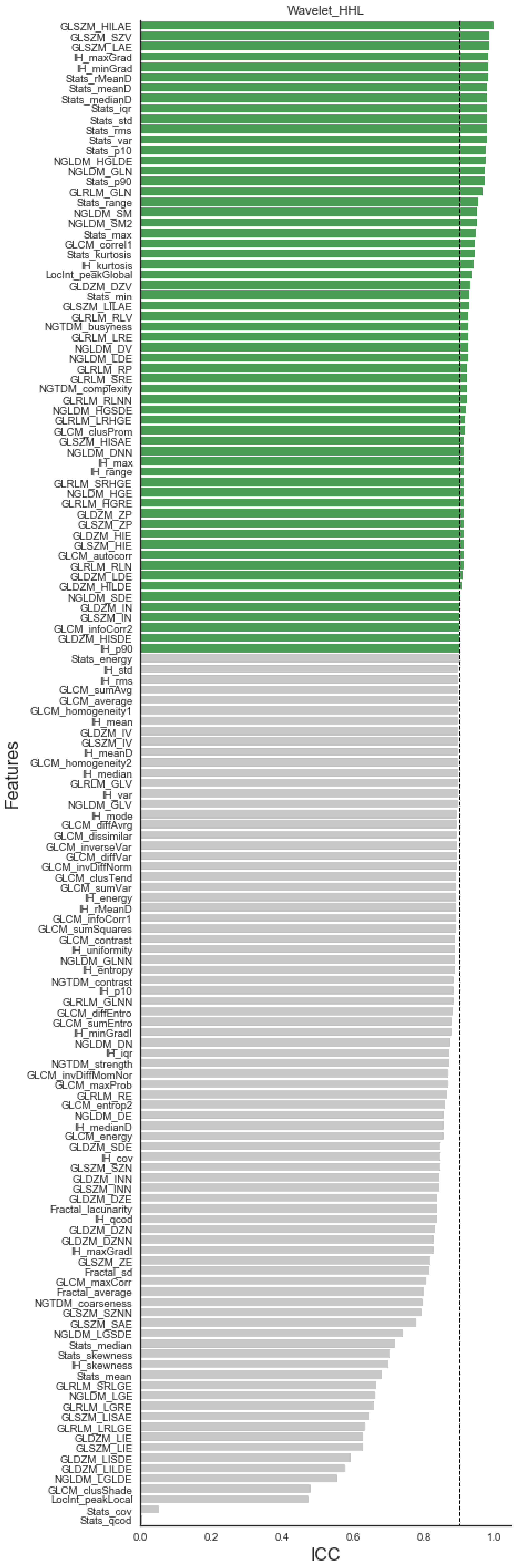

Wavelet_HHL
Features
ICC
GLSZM_HILAE
GLSZM_SZV
GLSZM_LAE
IH_maxGrad
IH_minGrad
Stats_rMeanD
Stats_meanD
Stats_medianD
Stats_iqr
Stats_std
Stats_rms
Stats_var
Stats_p10
NGLDM_HGLDE
NGLDM_GLN
Stats_p90
GLRLM_GLN
Stats_range
NGLDM_SM
NGLDM_SM2
Stats_max
GLCM_correl1
Stats_kurtosis
IH_kurtosis
LocInt_peakGlobal
GLDZM_DZV
Stats_min
GLSZM_LILAE
GLRLM_RLV
NGTDM_busyness
GLRLM_LRE
NGLDM_DV
NGLDM_LDE
GLRLM_RP
GLRLM_SRE
NGTDM_complexity
GLRLM_RLNN
NGLDM_HGSDE
GLRLM_LRHGE
GLCM_clusProm
GLSZM_HISAE
NGLDM_DNN
IH_max
IH_range
GLRLM_SRHGE
NGLDM_HGE
GLRLM_HGRE
GLDZM_ZP
GLSZM_ZP
GLDZM_HIE
GLSZM_HIE
GLCM_autocorr
GLRLM_RLN
GLDZM_LDE
GLDZM_HILDE
NGLDM_SDE
GLDZM_IN
GLSZM_IN
GLCM_infoCorr2
GLDZM_HISDE
IH_p90
Stats_energy
IH_std
IH_rms
GLCM_sumAvg
GLCM_average
GLCM_homogeneity1
IH_mean
GLDZM_IV
GLSZM_IV
IH_meanD
GLCM_homogeneity2
IH_median
GLRLM_GLV
IH_var
NGLDM_GLV
IH_mode
GLCM_diffAvrg
GLCM_dissimilar
GLCM_inverseVar
GLCM_diffVar
GLCM_invDiffNorm
GLCM_clusTend
GLCM_sumVar
IH_energy
IH_rMeanD
GLCM_infoCorr1
GLCM_sumSquares
GLCM_contrast
IH_uniformity
NGLDM_GLNN
IH_entropy
NGTDM_contrast
IH_p10
GLRLM_GLNN
GLCM_diffEntro
GLCM_sumEntro
IH_minGradI
NGLDM_DN
IH_iqr
NGTDM_strength
GLCM_invDiffMomNor
GLCM_maxProb
GLRLM_RE
GLCM_entrop2
NGLDM_DE
IH_medianD
GLCM_energy
GLDZM_SDE
IH_cov
GLSZM_SZN
GLDZM_INN
GLSZM_INN
GLDZM_DZE
Fractal_lacunarity
IH_qcod
GLDZM_DZN
GLDZM_DZNN
IH_maxGradI
GLSZM_ZE
Fractal_sd
GLCM_maxCorr
Fractal_average
NGTDM_coarseness
GLSZM_SZNN
GLSZM_SAE
NGLDM_LGSDE
Stats_median
Stats_skewness
IH_skewness
Stats_mean
GLRLM_SRLGE
NGLDM_LGE
GLRLM_LGRE
GLSZM_LISAE
GLRLM_LRLGE
GLDZM_LIE
GLSZM_LIE
GLDZM_LISDE
GLDZM_LILDE
NGLDM_LGLDE
GLCM_clusShade
LocInt_peakLocal
Stats_cov
Stats_qcod
0.0
0.2
0.4
0.6
0.8
1.0

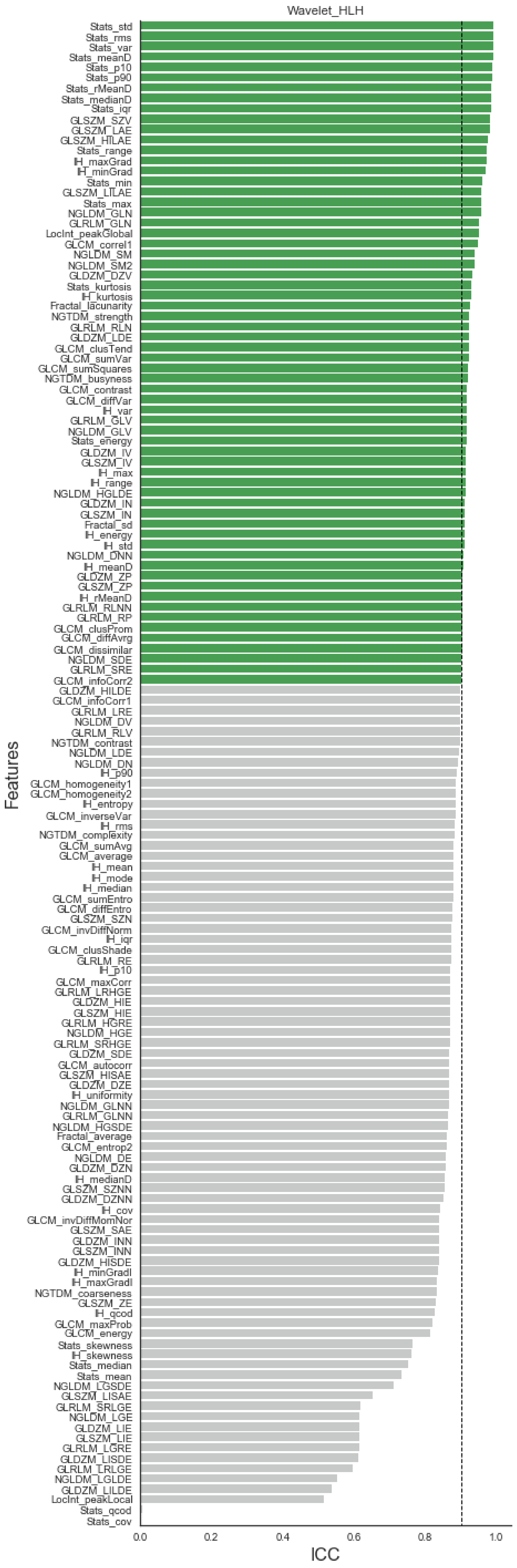
Wavelet_HLH
Features
ICC
Stats_std
Stats_rms
Stats_var
Stats_meanD
Stats_p10
Stats_p90
Stats_rMeanD
Stats_medianD
Stats_iqr
GLSZM_SZV
GLSZM_LAE
GLSZM_HILAE
Stats_range
IH_maxGrad
IH_minGrad
Stats_min
GLSZM_LILAE
Stats_max
NGLDM_GLN
GLRLM_GLN
LocInt_peakGlobal
GLCM_correl1
NGLDM_SM
NGLDM_SM2
GLDZM_DZV
Stats_kurtosis
IH_kurtosis
Fractal_lacunarity
NGTDM_strength
GLRLM_RLN
GLDZM_LDE
GLCM_clusTend
GLCM_sumVar
GLCM_sumSquares
NGTDM_busyness
GLCM_contrast
GLCM_diffVar
IH_var
GLRLM_GLV
NGLDM_GLV
Stats_energy
GLDZM_IV
GLSZM_IV
IH_max
IH_range
NGLDM_HGLDE
GLDZM_IN
GLSZM_IN
Fractal_sd
IH_energy
IH_std
NGLDM_DNN
IH_meanD
GLDZM_ZP
GLSZM_ZP
IH_rMeanD
GLRLM_RLNN
GLRLM_RP
GLCM_clusProm
GLCM_diffAvrg
GLCM_dissimilar
NGLDM_SDE
GLRLM_SRE
GLCM_infoCorr2
GLDZM_HILDE
GLCM_infoCorr1
GLRLM_LRE
NGLDM_DV
GLRLM_RLV
NGTDM_contrast
NGLDM_LDE
NGLDM_DN
IH_p90
GLCM_homogeneity1
GLCM_homogeneity2
IH_entropy
GLCM_inverseVar
IH_rms
NGTDM_complexity
GLCM_sumAvg
GLCM_average
IH_mean
IH_mode
IH_median
GLCM_sumEntro
GLCM_diffEntro
GLSZM_SZN
GLCM_invDiffNorm
IH_iqr
GLCM_clusShade
GLRLM_RE
IH_p10
GLCM_maxCorr
GLRLM_LRHGE
GLDZM_HIE
GLSZM_HIE
GLRLM_HGRE
NGLDM_HGE
GLRLM_SRHGE
GLDZM_SDE
GLCM_autocorr
GLSZM_HISAE
GLDZM_DZE
IH_uniformity
NGLDM_GLNN
GLRLM_GLNN
NGLDM_HGSDE
Fractal_average
GLCM_entrop2
NGLDM_DE
GLDZM_DZN
IH_medianD
GLSZM_SZNN
GLDZM_DZNN
IH_cov
GLCM_invDiffMomNor
GLSZM_SAE
GLDZM_INN
GLSZM_INN
GLDZM_HISDE
IH_minGradI
IH_maxGradI
NGTDM_coarseness
GLSZM_ZE
IH_qcod
GLCM_maxProb
GLCM_energy
Stats_skewness
IH_skewness
Stats_median
Stats_mean
NGLDM_LGSDE
GLSZM_LISAE
GLRLM_SRLGE
NGLDM_LGE
GLDZM_LIE
GLSZM_LIE
GLRLM_LGRE
GLDZM_LISDE
GLRLM_LRLGE
NGLDM_LGLDE
GLDZM_LILDE
LocInt_peakLocal
Stats_qcod
Stats_cov
0.0
0.2
0.4
0.6
0.8
1.0

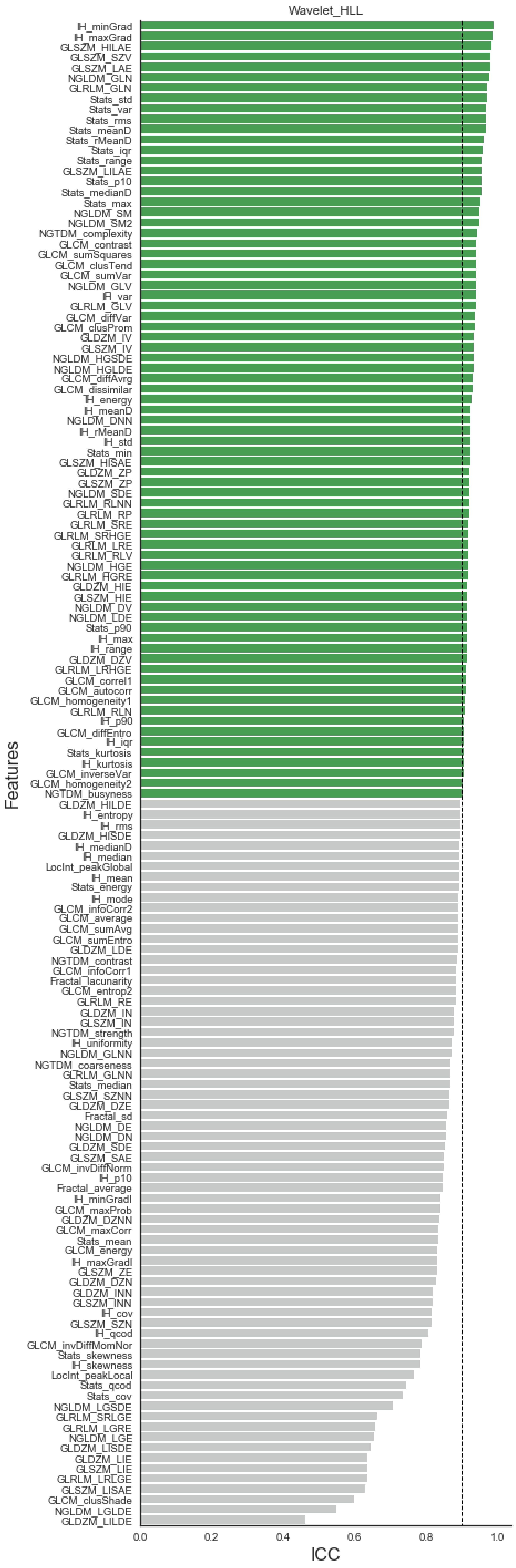
Wavelet_HLL
Features
ICC
0.0
0.2
0.4
0.6
0.8
1.0
IH_minGrad
IH_maxGrad
GLSZM_HILAE
GLSZM_SZV
GLSZM_LAE
NGLDM_GLN
GLRLM_GLN
Stats_std
Stats_var
Stats_rms
Stats_meanD
Stats_rMeanD
Stats_iqr
Stats_range
GLSZM_LILAE
Stats_p10
Stats_medianD
Stats_max
NGLDM_SM
NGLDM_SM2
NGTDM_complexity
GLCM_contrast
GLCM_sumSquares
GLCM_clusTend
GLCM_sumVar
NGLDM_GLV
IH_var
GLRLM_GLV
GLCM_diffVar
GLCM_clusProm
GLDZM_IV
GLSZM_IV
NGLDM_HGSDE
NGLDM_HGLDE
GLCM_diffAvrg
GLCM_dissimilar
IH_energy
IH_meanD
NGLDM_DNN
IH_rMeanD
IH_std
Stats_min
GLSZM_HISAE
GLDZM_ZP
GLSZM_ZP
NGLDM_SDE
GLRLM_RLNN
GLRLM_RP
GLRLM_SRE
GLRLM_SRHGE
GLRLM_LRE
GLRLM_RLV
NGLDM_HGE
GLRLM_HGRE
GLDZM_HIE
GLSZM_HIE
NGLDM_DV
NGLDM_LDE
Stats_p90
IH_max
IH_range
GLDZM_DZV
GLRLM_LRHGE
GLCM_correl1
GLCM_autocorr
GLCM_homogeneity1
GLRLM_RLN
IH_p90
GLCM_diffEntro
IH_iqr
Stats_kurtosis
IH_kurtosis
GLCM_inverseVar
GLCM_homogeneity2
NGTDM_busyness
GLDZM_HILDE
IH_entropy
IH_rms
GLDZM_HISDE
IH_medianD
IH_median
LocInt_peakGlobal
IH_mean
Stats_energy
IH_mode
GLCM_infoCorr2
GLCM_average
GLCM_sumAvg
GLCM_sumEntro
GLDZM_LDE
NGTDM_contrast
GLCM_infoCorr1
Fractal_lacunarity
GLCM_entrop2
GLRLM_RE
GLDZM_IN
GLSZM_IN
NGTDM_strength
IH_uniformity
NGLDM_GLNN
NGTDM_coarseness
GLRLM_GLNN
Stats_median
GLSZM_SZNN
GLDZM_DZE
Fractal_sd
NGLDM_DE
NGLDM_DN
GLDZM_SDE
GLSZM_SAE
GLCM_invDiffNorm
IH_p10
Fractal_average
IH_minGradI
GLCM_maxProb
GLDZM_DZNN
GLCM_maxCorr
Stats_mean
GLCM_energy
IH_maxGradI
GLSZM_ZE
GLDZM_DZN
GLDZM_INN
GLSZM_INN
IH_cov
GLSZM_SZN
IH_qcod
GLCM_invDiffMomNor
Stats_skewness
IH_skewness
LocInt_peakLocal
Stats_qcod
Stats_cov
NGLDM_LGSDE
GLRLM_SRLGE
GLRLM_LGRE
NGLDM_LGE
GLDZM_LISDE
GLDZM_LIE
GLSZM_LIE
GLRLM_LRLGE
GLSZM_LISAE
GLCM_clusShade
NGLDM_LGLDE
GLDZM_LILDE

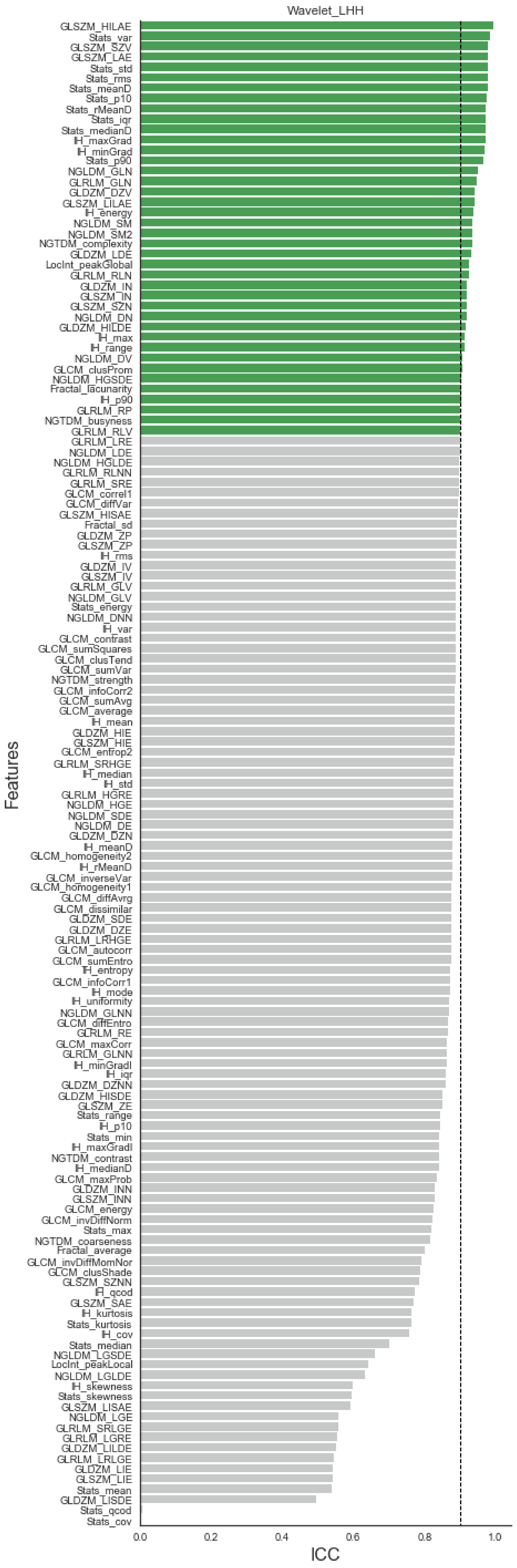
Wavelet_LHH
Features
ICC
GLSZM_HILAE
Stats_var
GLSZM_SZV
GLSZM_LAE
Stats_std
Stats_rms
Stats_meanD
Stats_p10
Stats_rMeanD
Stats_iqr
Stats_medianD
IH_maxGrad
IH_minGrad
Stats_p90
NGLDM_GLN
GLRLM_GLN
GLDZM_DZV
GLSZM_LILAE
IH_energy
NGLDM_SM
NGLDM_SM2
NGTDM_complexity
GLDZM_LDE
LocInt_peakGlobal
GLRLM_RLN
GLDZM_IN
GLSZM_IN
GLSZM_SZN
NGLDM_DN
GLDZM_HILDE
IH_max
IH_range
NGLDM_DV
GLCM_clusProm
NGLDM_HGSDE
Fractal_lacunarity
IH_p90
GLRLM_RP
NGTDM_busyness
GLRLM_RLV
GLRLM_LRE
NGLDM_LDE
NGLDM_HGLDE
GLRLM_RLNN
GLRLM_SRE
GLCM_correl1
GLCM_diffVar
GLSZM_HISAE
Fractal_sd
GLDZM_ZP
GLSZM_ZP
IH_rms
GLDZM_IV
GLSZM_IV
GLRLM_GLV
NGLDM_GLV
Stats_energy
NGLDM_DNN
IH_var
GLCM_contrast
GLCM_sumSquares
GLCM_clusTend
GLCM_sumVar
NGTDM_strength
GLCM_infoCorr2
GLCM_sumAvg
GLCM_average
IH_mean
GLDZM_HIE
GLSZM_HIE
GLCM_entrop2
GLRLM_SRHGE
IH_median
IH_std
GLRLM_HGRE
NGLDM_HGE
NGLDM_SDE
NGLDM_DE
GLDZM_DZN
IH_meanD
GLCM_homogeneity2
IH_rMeanD
GLCM_inverseVar
GLCM_homogeneity1
GLCM_diffAvrg
GLCM_dissimilar
GLDZM_SDE
GLDZM_DZE
GLRLM_LRHGE
GLCM_autocorr
GLCM_sumEntro
IH_entropy
GLCM_infoCorr1
IH_mode
IH_uniformity
NGLDM_GLNN
GLCM_diffEntro
GLRLM_RE
GLCM_maxCorr
GLRLM_GLNN
IH_minGradI
IH_iqr
GLDZM_DZNN
GLDZM_HISDE
GLSZM_ZE
Stats_range
IH_p10
Stats_min
IH_maxGradI
NGTDM_contrast
IH_medianD
GLCM_maxProb
GLDZM_INN
GLSZM_INN
GLCM_energy
GLCM_invDiffNorm
Stats_max
NGTDM_coarseness
Fractal_average
GLCM_invDiffMomNor
GLCM_clusShade
GLSZM_SZNN
IH_qcod
GLSZM_SAE
IH_kurtosis
Stats_kurtosis
IH_cov
Stats_median
NGLDM_LGSDE
LocInt_peakLocal
NGLDM_LGLDE
IH_skewness
Stats_skewness
GLSZM_LISAE
NGLDM_LGE
GLRLM_SRLGE
GLRLM_LGRE
GLDZM_LILDE
GLRLM_LRLGE
GLDZM_LIE
GLSZM_LIE
Stats_mean
GLDZM_LISDE
Stats_qcod
Stats_cov
0.0
0.2
0.4
0.6
0.8
1.0

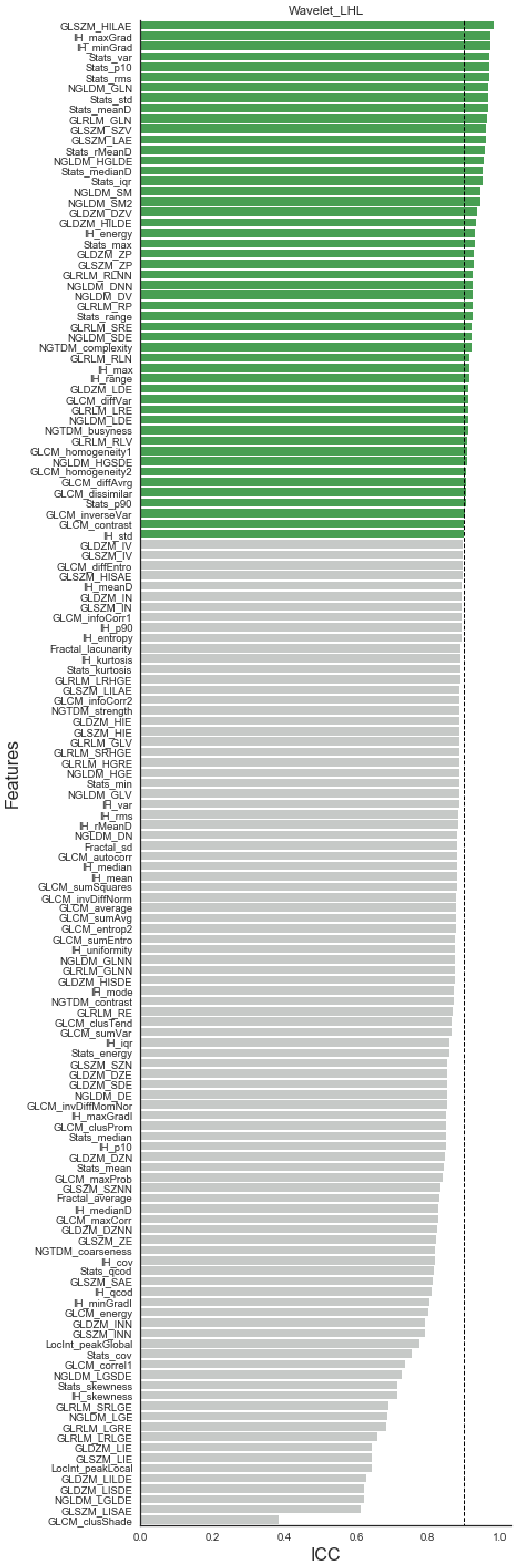

Wavelet_LHL
Features
ICC
GLSZM_HILAE
IH_maxGrad
IH_minGrad
Stats_var
Stats_p10
Stats_rms
NGLDM_GLN
Stats_std
Stats_meanD
GLRLM_GLN
GLSZM_SZV
GLSZM_LAE
Stats_rMeanD
NGLDM_HGLDE
Stats_medianD
Stats_iqr
NGLDM_SM
NGLDM_SM2
GLDZM_DZV
GLDZM_HILDE
IH_energy
Stats_max
GLDZM_ZP
GLSZM_ZP
GLRLM_RLNN
NGLDM_DNN
NGLDM_DV
GLRLM_RP
Stats_range
GLRLM_SRE
NGLDM_SDE
NGTDM_complexity
GLRLM_RLN
IH_max
IH_range
GLDZM_LDE
GLCM_diffVar
GLRLM_LRE
NGLDM_LDE
NGTDM_busyness
GLRLM_RLV
GLCM_homogeneity1
NGLDM_HGSDE
GLCM_homogeneity2
GLCM_diffAvrg
GLCM_dissimilar
Stats_p90
GLCM_inverseVar
GLCM_contrast
IH_std
GLDZM_IV
GLSZM_IV
GLCM_diffEntro
GLSZM_HISAE
IH_meanD
GLDZM_IN
GLSZM_IN
GLCM_infoCorr1
IH_p90
IH_entropy
Fractal_lacunarity
IH_kurtosis
Stats_kurtosis
GLRLM_LRHGE
GLSZM_LILAE
GLCM_infoCorr2
NGTDM_strength
GLDZM_HIE
GLSZM_HIE
GLRLM_GLV
GLRLM_SRHGE
GLRLM_HGRE
NGLDM_HGE
Stats_min
NGLDM_GLV
IH_var
IH_rms
IH_rMeanD
NGLDM_DN
Fractal_sd
GLCM_autocorr
IH_median
IH_mean
GLCM_sumSquares
GLCM_invDiffNorm
GLCM_average
GLCM_sumAvg
GLCM_entrop2
GLCM_sumEntro
IH_uniformity
NGLDM_GLNN
GLRLM_GLNN
GLDZM_HISDE
IH_mode
NGTDM_contrast
GLRLM_RE
GLCM_clusTend
GLCM_sumVar
IH_iqr
Stats_energy
GLSZM_SZN
GLDZM_DZE
GLDZM_SDE
NGLDM_DE
GLCM_invDiffMomNor
IH_maxGradl
GLCM_clusProm
Stats_median
IH_p10
GLDZM_DZN
Stats_mean
GLCM_maxProb
GLSZM_SZNN
Fractal_average
IH_medianD
GLCM_maxCorr
GLDZM_DZNN
GLSZM_ZE
NGTDM_coarseness
IH_cov
Stats_qcod
GLSZM_SAE
IH_qcod
IH_minGradl
GLCM_energy
GLDZM_INN
GLSZM_INN
LocInt_peakGlobal
Stats_cov
GLCM_correl1
NGLDM_LGSDE
Stats_skewness
IH_skewness
GLRLM_SRLGE
NGLDM_LGE
GLRLM_LGRE
GLRLM_LRLGE
GLDZM_LIE
GLSZM_LIE
LocInt_peakLocal
GLDZM_LILDE
GLDZM_LISDE
NGLDM_LGLDE
GLSZM_LISAE
GLCM_clusShade
0.0
0.2
0.4
0.6
0.8
1.0

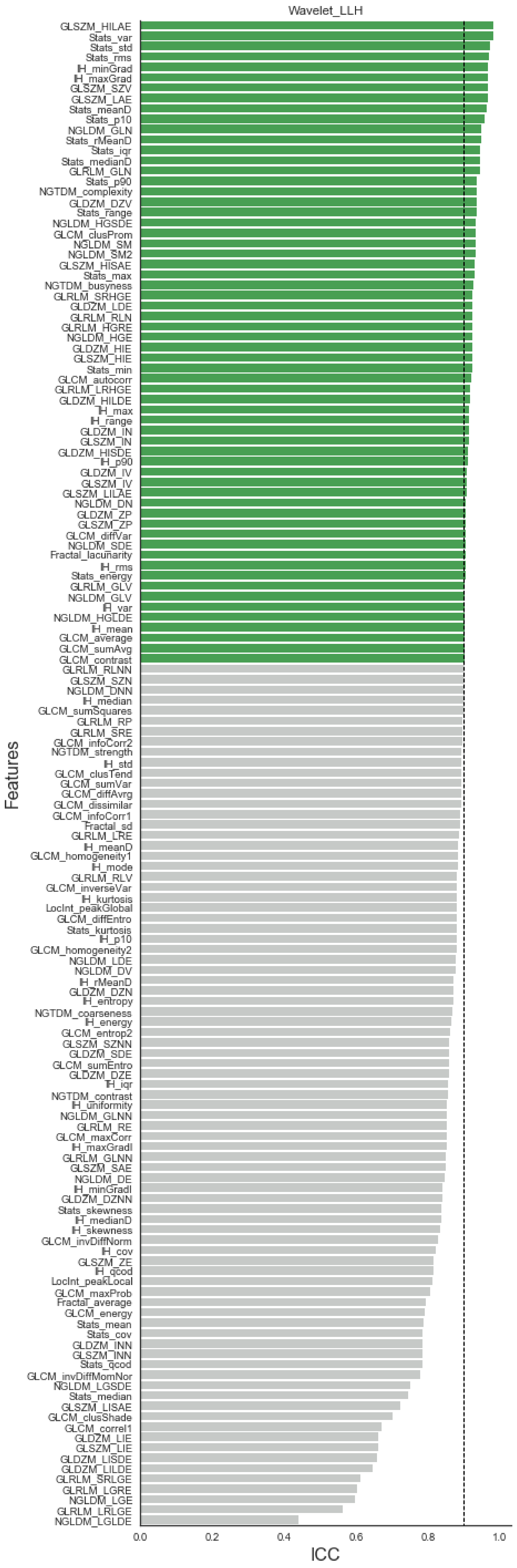

Wavelet_LLH
Features
ICC
GLSZM_HILAE
Stats_var
Stats_std
Stats_rms
IH_minGrad
IH_maxGrad
GLSZM_SZV
GLSZM_LAE
Stats_meanD
Stats_p10
NGLDM_GLN
Stats_rMeanD
Stats_iqr
Stats_medianD
GLRLM_GLN
Stats_p90
NGTDM_complexity
GLDZM_DZV
Stats_range
NGLDM_HGSDE
GLCM_clusProm
NGLDM_SM
NGLDM_SM2
GLSZM_HISAE
Stats_max
NGTDM_busyness
GLRLM_SRHGE
GLDZM_LDE
GLRLM_RLN
GLRLM_HGRE
NGLDM_HGE
GLDZM_HIE
GLSZM_HIE
Stats_min
GLCM_autocorr
GLRLM_LRHGE
GLDZM_HILDE
IH_max
IH_range
GLDZM_IN
GLSZM_IN
GLDZM_HISDE
IH_p90
GLDZM_IV
GLSZM_IV
GLSZM_LILAE
NGLDM_DN
GLDZM_ZP
GLSZM_ZP
GLCM_diffVar
NGLDM_SDE
Fractal_lacunarity
IH_rms
Stats_energy
GLRLM_GLV
NGLDM_GLV
IH_var
NGLDM_HGLDE
IH_mean
GLCM_average
GLCM_sumAvg
GLCM_contrast
GLRLM_RLNN
GLSZM_SZN
NGLDM_DNN
IH_median
GLCM_sumSquares
GLRLM_RP
GLRLM_SRE
GLCM_infoCorr2
NGTDM_strength
IH_std
GLCM_clusTend
GLCM_sumVar
GLCM_diffAvrg
GLCM_dissimilar
GLCM_infoCorr1
Fractal_sd
GLRLM_LRE
IH_meanD
GLCM_homogeneity1
IH_mode
GLRLM_RLV
GLCM_inverseVar
IH_kurtosis
LocInt_peakGlobal
GLCM_diffEntro
Stats_kurtosis
IH_p10
GLCM_homogeneity2
NGLDM_LDE
NGLDM_DV
IH_rMeanD
GLDZM_DZN
IH_entropy
NGTDM_coarseness
IH_energy
GLCM_entrop2
GLSZM_SZNN
GLDZM_SDE
GLCM_sumEntro
GLDZM_DZE
IH_iqr
NGTDM_contrast
IH_uniformity
NGLDM_GLNN
GLRLM_RE
GLCM_maxCorr
IH_maxGradI
GLRLM_GLNN
GLSZM_SAE
NGLDM_DE
IH_minGradI
GLDZM_DZNN
Stats_skewness
IH_medianD
IH_skewness
GLCM_invDiffNorm
IH_cov
GLSZM_ZE
IH_qcod
LocInt_peakLocal
GLCM_maxProb
Fractal_average
GLCM_energy
Stats_mean
Stats_cov
GLDZM_INN
GLSZM_INN
Stats_qcod
GLCM_invDiffMomNor
NGLDM_LGSDE
Stats_median
GLSZM_LISAE
GLCM_clusShade
GLCM_correl1
GLDZM_LIE
GLSZM_LIE
GLDZM_LISDE
GLDZM_LILDE
GLRLM_SRLGE
GLRLM_LGRE
NGLDM_LGE
GLRLM_LRLGE
NGLDM_LGLDE
0.0
0.2
0.4
0.6
0.8
1.0

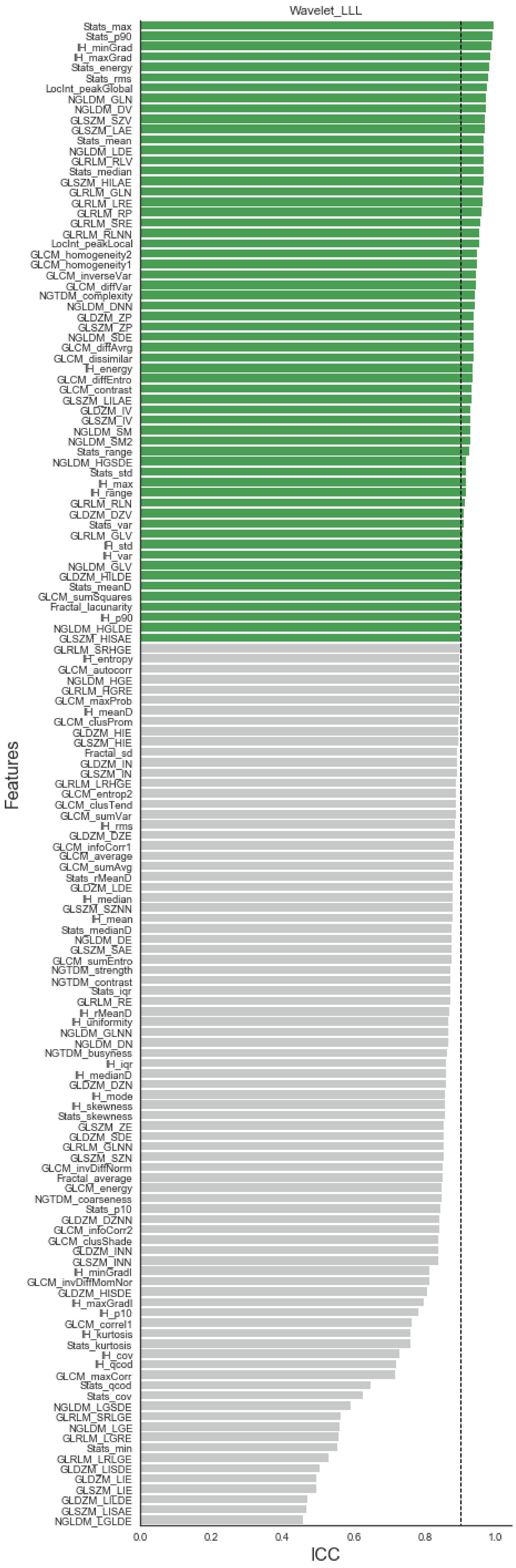

Wavelet_LLL
Features
ICC
Stats_max
Stats_p90
IH_minGrad
IH_maxGrad
Stats_energy
Stats_rms
LocInt_peakGlobal
NGLDM_GLN
NGLDM_DV
GLSZM_SZV
GLSZM_LAE
Stats_mean
NGLDM_LDE
GLRLM_RLV
Stats_median
GLSZM_HILAE
GLRLM_GLN
GLRLM_LRE
GLRLM_RP
GLRLM_SRE
GLRLM_RLNN
LocInt_peakLocal
GLCM_homogeneity2
GLCM_homogeneity1
GLCM_inverseVar
GLCM_diffVar
NGTDM_complexity
NGLDM_DNN
GLDZM_ZP
GLSZM_ZP
NGLDM_SDE
GLCM_diffAvrg
GLCM_dissimilar
IH_energy
GLCM_diffEntro
GLCM_contrast
GLSZM_LILAE
GLDZM_IV
GLSZM_IV
NGLDM_SM
NGLDM_SM2
Stats_range
NGLDM_HGSDE
Stats_std
IH_max
IH_range
GLRLM_RLN
GLDZM_DZV
Stats_var
GLRLM_GLV
IH_std
IH_var
NGLDM_GLV
GLDZM_HILDE
Stats_meanD
GLCM_sumSquares
Fractal_lacunarity
IH_p90
NGLDM_HGLDE
GLSZM_HISAE
GLRLM_SRHGE
IH_entropy
GLCM_autocorr
NGLDM_HGE
GLRLM_HGRE
GLCM_maxProb
IH_meanD
GLCM_clusProm
GLDZM_HIE
GLSZM_HIE
Fractal_sd
GLDZM_IN
GLSZM_IN
GLRLM_LRHGE
GLCM_entrop2
GLCM_clusTend
GLCM_sumVar
IH_rms
GLDZM_DZE
GLCM_infoCorr1
GLCM_average
GLCM_sumAvg
Stats_rMeanD
GLDZM_LDE
IH_median
GLSZM_SZNN
IH_mean
Stats_medianD
NGLDM_DE
GLSZM_SAE
GLCM_sumEntro
NGTDM_strength
NGTDM_contrast
Stats_iqr
GLRLM_RE
IH_rMeanD
IH_uniformity
NGLDM_GLNN
NGLDM_DN
NGTDM_busyness
IH_iqr
IH_medianD
GLDZM_DZN
IH_mode
IH_skewness
Stats_skewness
GLSZM_ZE
GLDZM_SDE
GLRLM_GLNN
GLSZM_SZN
GLCM_invDiffNorm
Fractal_average
GLCM_energy
NGTDM_coarseness
Stats_p10
GLDZM_DZNN
GLCM_infoCorr2
GLCM_clusShade
GLDZM_INN
GLSZM_INN
IH_minGradI
GLCM_invDiffMomNor
GLDZM_HISDE
IH_maxGradI
IH_p10
GLCM_correl1
IH_kurtosis
Stats_kurtosis
IH_cov
IH_qcod
GLCM_maxCorr
Stats_qcod
Stats_cov
NGLDM_LGSDE
GLRLM_SRLGE
NGLDM_LGE
GLRLM_LGRE
Stats_min
GLRLM_LRLGE
GLDZM_LISDE
GLDZM_LIE
GLSZM_LIE
GLDZM_LILDE
GLSZM_LISAE
NGLDM_LGLDE
0.0
0.2
0.4
0.6
0.8
1.0

Supplementary material 2. ICC values of all wavelet Pyradiomics features per wavelet decomposition with robust features (ICC > 0.9) shown in green

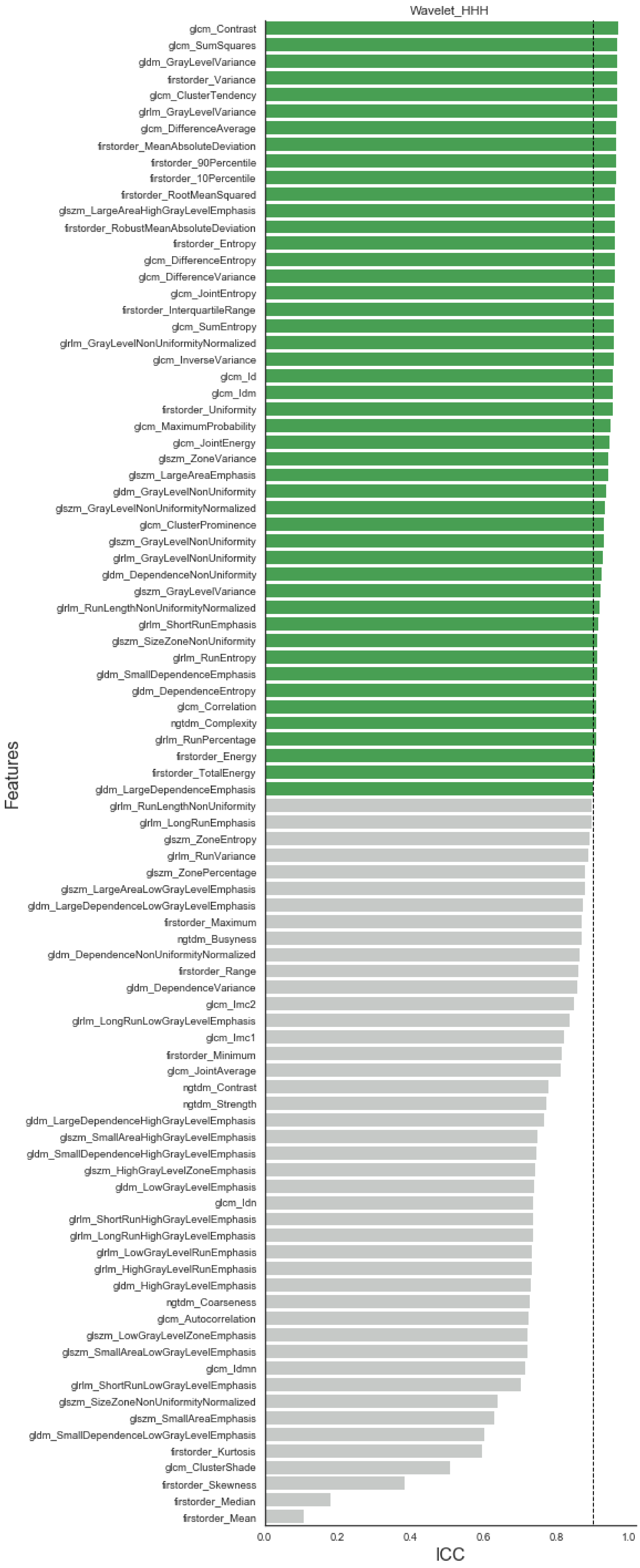
Wavelet_HHH
glcm_Contrast
glcm_SumSquares
gldm_GrayLevelVariance
firstorder_Variance
glcm_ClusterTendency
glrlm_GrayLevelVariance
glcm_DifferenceAverage
firstorder_MeanAbsoluteDeviation
firstorder_90Percentile
firstorder_10Percentile
firstorder_RootMeanSquared
glszm_LargeAreaHighGrayLevelEmphasis
firstorder_RobustMeanAbsoluteDeviation
firstorder_Entropy
glcm_DifferenceEntropy
glcm_DifferenceVariance
glcm_JointEntropy
firstorder_InterquartileRange
glcm_SumEntropy
glrlm_GrayLevelNonUniformityNormalized
glcm_InverseVariance
glcm_Id
glcm_Idm
firstorder_Uniformity
glcm_MaximumProbability
glcm_JointEnergy
glszm_ZoneVariance
glszm_LargeAreaEmphasis
gldm_GrayLevelNonUniformity
glszm_GrayLevelNonUniformityNormalized
glcm_ClusterProminence
glszm_GrayLevelNonUniformity
glrlm_GrayLevelNonUniformity
gldm_DependenceNonUniformity
glszm_GrayLevelVariance
glrlm_RunLengthNonUniformityNormalized
glrlm_ShortRunEmphasis
glszm_SizeZoneNonUniformity
glrlm_RunEntropy
gldm_SmallDependenceEmphasis
gldm_DependenceEntropy
glcm_Correlation
ngtdm_Complexity
glrlm_RunPercentage
firstorder_Energy
firstorder_TotalEnergy
gldm_LargeDependenceEmphasis
glrlm_RunLengthNonUniformity
glrlm_LongRunEmphasis
glszm_ZoneEntropy
glrlm_RunVariance
glszm_ZonePercentage
glszm_LargeAreaLowGrayLevelEmphasis
gldm_LargeDependenceLowGrayLevelEmphasis
firstorder_Maximum
ngtdm_Busyness
gldm_DependenceNonUniformityNormalized
firstorder_Range
gldm_DependenceVariance
glcm_Imc2
glrlm_LongRunLowGrayLevelEmphasis
glcm_Imc1
firstorder_Minimum
glcm_JointAverage
ngtdm_Contrast
ngtdm_Strength
gldm_LargeDependenceHighGrayLevelEmphasis
glszm_SmallAreaHighGrayLevelEmphasis
gldm_SmallDependenceHighGrayLevelEmphasis
glszm_HighGrayLevelZoneEmphasis
gldm_LowGrayLevelEmphasis
glcm_Idn
glrlm_ShortRunHighGrayLevelEmphasis
glrlm_LongRunHighGrayLevelEmphasis
glrlm_LowGrayLevelRunEmphasis
glrlm_HighGrayLevelRunEmphasis
gldm_HighGrayLevelEmphasis
ngtdm_Coarseness
glcm_Autocorrelation
glszm_LowGrayLevelZoneEmphasis
glszm_SmallAreaLowGrayLevelEmphasis
glcm_Idmn
glrlm_ShortRunLowGrayLevelEmphasis
glszm_SizeZoneNonUniformityNormalized
glszm_SmallAreaEmphasis
gldm_SmallDependenceLowGrayLevelEmphasis
firstorder_Kurtosis
glcm_ClusterShade
firstorder_Skewness
firstorder_Median
firstorder_Mean
Features
0.0
0.2
0.4
0.6
0.8
1.0
ICC

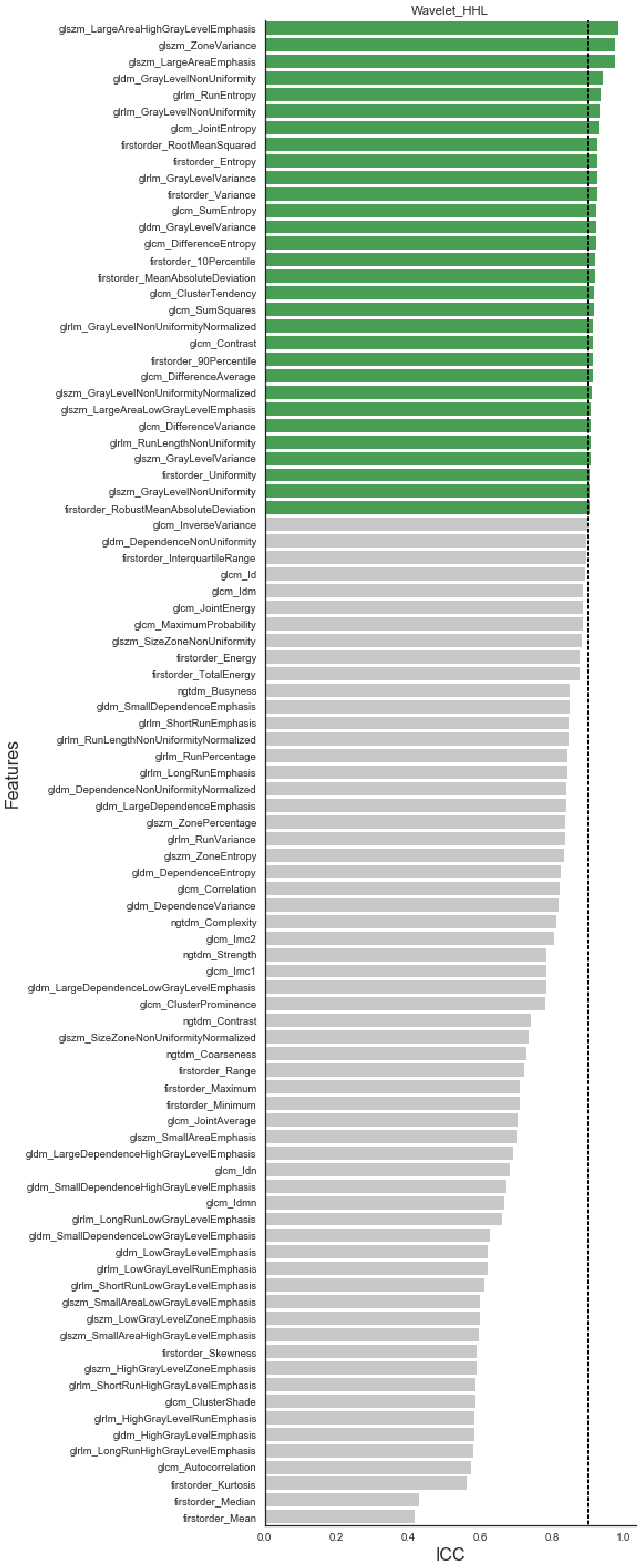

Wavelet_HHL
glszm_LargeAreaHighGrayLevelEmphasis
glszm_ZoneVariance
glszm_LargeAreaEmphasis
gldm_GrayLevelNonUniformity
glrlm_RunEntropy
glrlm_GrayLevelNonUniformity
glcm_JointEntropy
firstorder_RootMeanSquared
firstorder_Entropy
glrlm_GrayLevelVariance
firstorder_Variance
glcm_SumEntropy
gldm_GrayLevelVariance
glcm_DifferenceEntropy
firstorder_10Percentile
firstorder_MeanAbsoluteDeviation
glcm_ClusterTendency
glcm_SumSquares
glrlm_GrayLevelNonUniformityNormalized
glcm_Contrast
firstorder_90Percentile
glcm_DifferenceAverage
glszm_GrayLevelNonUniformityNormalized
glszm_LargeAreaLowGrayLevelEmphasis
glcm_DifferenceVariance
glrlm_RunLengthNonUniformity
glszm_GrayLevelVariance
firstorder_Uniformity
glszm_GrayLevelNonUniformity
firstorder_RobustMeanAbsoluteDeviation
glcm_InverseVariance
gldm_DependenceNonUniformity
firstorder_InterquartileRange
glcm_Id
glcm_Idm
glcm_JointEnergy
glcm_MaximumProbability
glszm_SizeZoneNonUniformity
firstorder_Energy
firstorder_TotalEnergy
ngtdm_Busyness
gldm_SmallDependenceEmphasis
glrlm_ShortRunEmphasis
glrlm_RunLengthNonUniformityNormalized
glrlm_RunPercentage
glrlm_LongRunEmphasis
gldm_DependenceNonUniformityNormalized
gldm_LargeDependenceEmphasis
glszm_ZonePercentage
glrlm_RunVariance
glszm_ZoneEntropy
gldm_DependenceEntropy
glcm_Correlation
gldm_DependenceVariance
ngtdm_Complexity
glcm_Imc2
ngtdm_Strength
glcm_Imc1
gldm_LargeDependenceLowGrayLevelEmphasis
glcm_ClusterProminence
ngtdm_Contrast
glszm_SizeZoneNonUniformityNormalized
ngtdm_Coarseness
firstorder_Range
firstorder_Maximum
firstorder_Minimum
glcm_JointAverage
glszm_SmallAreaEmphasis
gldm_LargeDependenceHighGrayLevelEmphasis
glcm_Idn
gldm_SmallDependenceHighGrayLevelEmphasis
glcm_Idmn
glrlm_LongRunLowGrayLevelEmphasis
gldm_SmallDependenceLowGrayLevelEmphasis
gldm_LowGrayLevelEmphasis
glrlm_LowGrayLevelRunEmphasis
glrlm_ShortRunLowGrayLevelEmphasis
glszm_SmallAreaLowGrayLevelEmphasis
glszm_LowGrayLevelZoneEmphasis
glszm_SmallAreaHighGrayLevelEmphasis
firstorder_Skewness
glszm_HighGrayLevelZoneEmphasis
glrlm_ShortRunHighGrayLevelEmphasis
glcm_ClusterShade
glrlm_HighGrayLevelRunEmphasis
gldm_HighGrayLevelEmphasis
glrlm_LongRunHighGrayLevelEmphasis
glcm_Autocorrelation
firstorder_Kurtosis
firstorder_Median
firstorder_Mean
Features
0.0
0.2
0.4
0.6
0.8
1.0
ICC

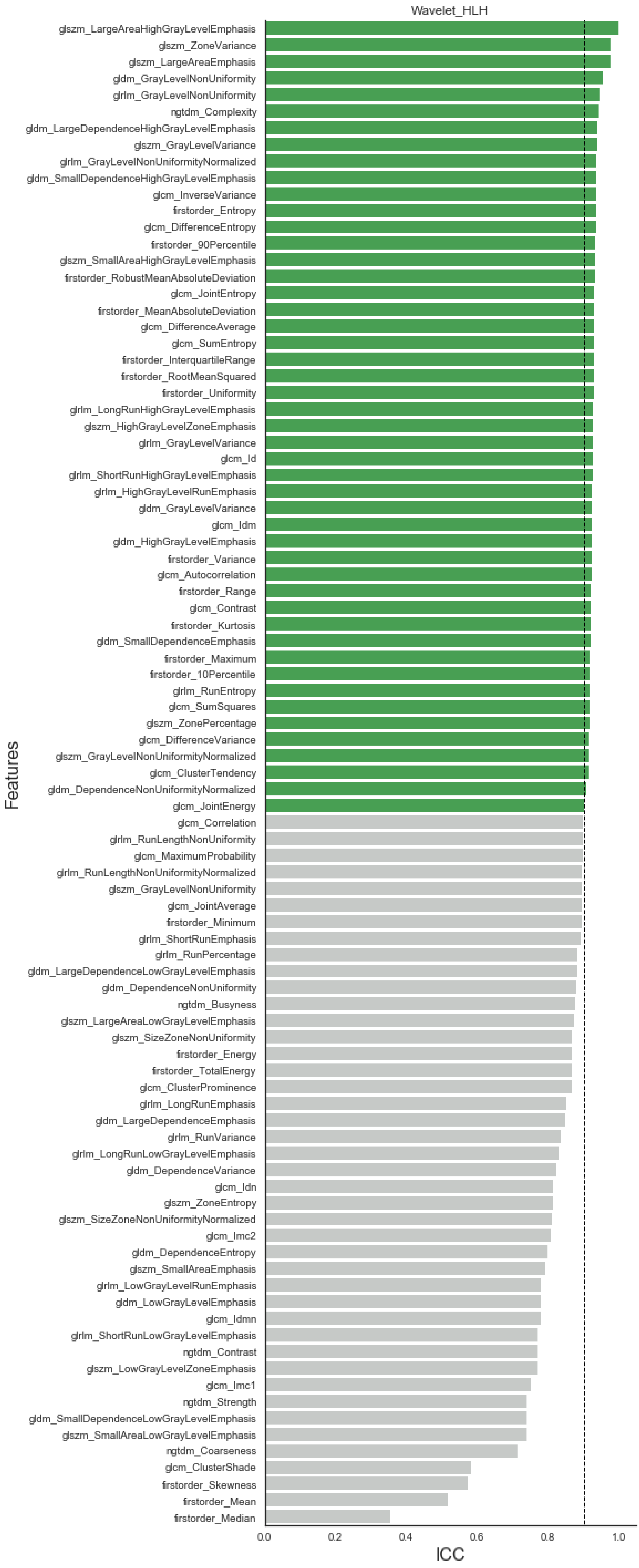
Wavelet_HLH
Features
ICC
0.0
0.2
0.4
0.6
0.8
1.0
glszm_LargeAreaHighGrayLevelEmphasis
glszm_ZoneVariance
glszm_LargeAreaEmphasis
gldm_GrayLevelNonUniformity
glrlm_GrayLevelNonUniformity
ngtdm_Complexity
gldm_LargeDependenceHighGrayLevelEmphasis
glszm_GrayLevelVariance
glrlm_GrayLevelNonUniformityNormalized
gldm_SmallDependenceHighGrayLevelEmphasis
glcm_InverseVariance
firstorder_Entropy
glcm_DifferenceEntropy
firstorder_90Percentile
glszm_SmallAreaHighGrayLevelEmphasis
firstorder_RobustMeanAbsoluteDeviation
glcm_JointEntropy
firstorder_MeanAbsoluteDeviation
glcm_DifferenceAverage
glcm_SumEntropy
firstorder_InterquartileRange
firstorder_RootMeanSquared
firstorder_Uniformity
glrlm_LongRunHighGrayLevelEmphasis
glszm_HighGrayLevelZoneEmphasis
glrlm_GrayLevelVariance
glcm_Id
glrlm_ShortRunHighGrayLevelEmphasis
glrlm_HighGrayLevelRunEmphasis
gldm_GrayLevelVariance
glcm_Idm
gldm_HighGrayLevelEmphasis
firstorder_Variance
glcm_Autocorrelation
firstorder_Range
glcm_Contrast
firstorder_Kurtosis
gldm_SmallDependenceEmphasis
firstorder_Maximum
firstorder_10Percentile
glrlm_RunEntropy
glcm_SumSquares
glszm_ZonePercentage
glcm_DifferenceVariance
glszm_GrayLevelNonUniformityNormalized
glcm_ClusterTendency
gldm_DependenceNonUniformityNormalized
glcm_JointEnergy
glcm_Correlation
glrlm_RunLengthNonUniformity
glcm_MaximumProbability
glrlm_RunLengthNonUniformityNormalized
glszm_GrayLevelNonUniformity
glcm_JointAverage
firstorder_Minimum
glrlm_ShortRunEmphasis
glrlm_RunPercentage
gldm_LargeDependenceLowGrayLevelEmphasis
gldm_DependenceNonUniformity
ngtdm_Busyness
glszm_LargeAreaLowGrayLevelEmphasis
glszm_SizeZoneNonUniformity
firstorder_Energy
firstorder_TotalEnergy
glcm_ClusterProminence
glrlm_LongRunEmphasis
gldm_LargeDependenceEmphasis
glrlm_RunVariance
glrlm_LongRunLowGrayLevelEmphasis
gldm_DependenceVariance
glcm_Idn
glszm_ZoneEntropy
glszm_SizeZoneNonUniformityNormalized
glcm_Imc2
gldm_DependenceEntropy
glszm_SmallAreaEmphasis
glrlm_LowGrayLevelRunEmphasis
gldm_LowGrayLevelEmphasis
glcm_Idmn
glrlm_ShortRunLowGrayLevelEmphasis
ngtdm_Contrast
glszm_LowGrayLevelZoneEmphasis
glcm_Imc1
ngtdm_Strength
gldm_SmallDependenceLowGrayLevelEmphasis
glszm_SmallAreaLowGrayLevelEmphasis
ngtdm_Coarseness
glcm_ClusterShade
firstorder_Skewness
firstorder_Mean
firstorder_Median

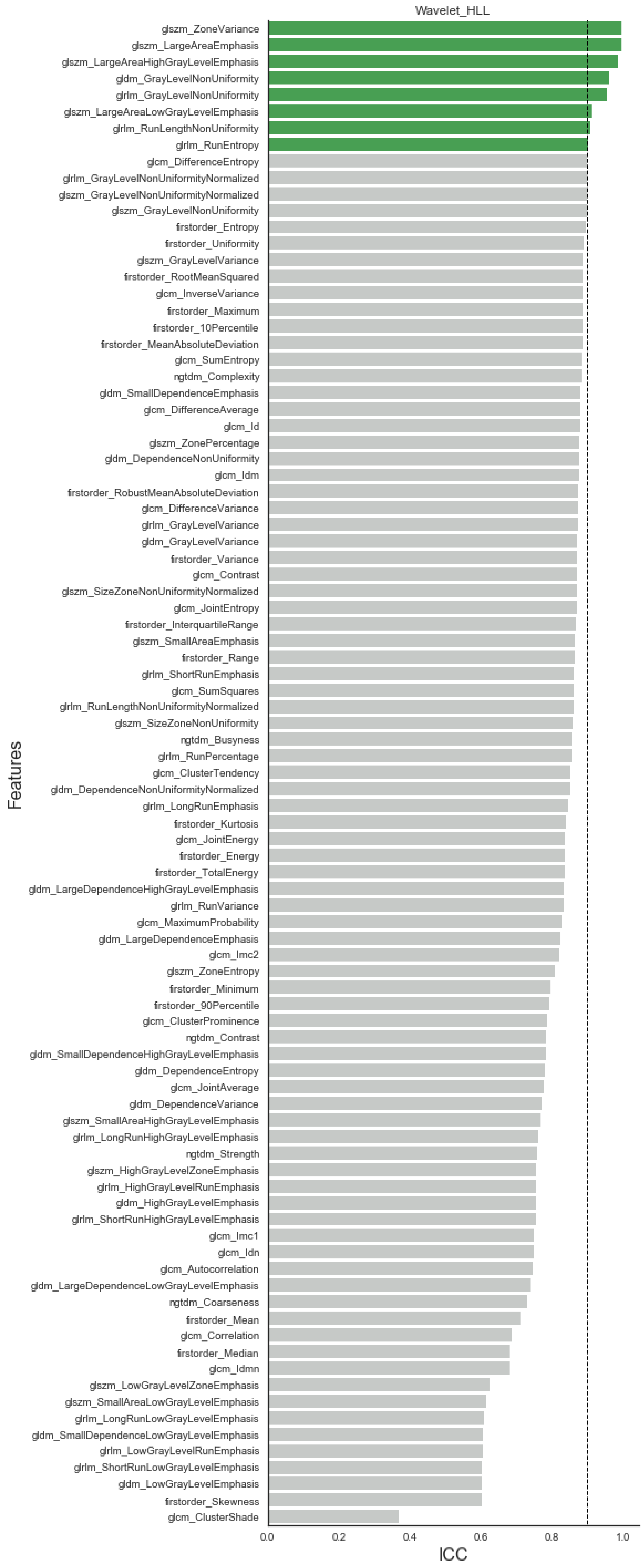
Wavelet_HLL
glszm_ZoneVariance
glszm_LargeAreaEmphasis
glszm_LargeAreaHighGrayLevelEmphasis
gldm_GrayLevelNonUniformity
glrlm_GrayLevelNonUniformity
glszm_LargeAreaLowGrayLevelEmphasis
glrlm_RunLengthNonUniformity
glrlm_RunEntropy
glcm_DifferenceEntropy
glrlm_GrayLevelNonUniformityNormalized
glszm_GrayLevelNonUniformityNormalized
glszm_GrayLevelNonUniformity
firstorder_Entropy
firstorder_Uniformity
glszm_GrayLevelVariance
firstorder_RootMeanSquared
glcm_InverseVariance
firstorder_Maximum
firstorder_10Percentile
firstorder_MeanAbsoluteDeviation
glcm_SumEntropy
ngtdm_Complexity
gldm_SmallDependenceEmphasis
glcm_DifferenceAverage
glcm_Id
glszm_ZonePercentage
gldm_DependenceNonUniformity
glcm_Idm
firstorder_RobustMeanAbsoluteDeviation
glcm_DifferenceVariance
glrlm_GrayLevelVariance
gldm_GrayLevelVariance
firstorder_Variance
glcm_Contrast
glszm_SizeZoneNonUniformityNormalized
glcm_JointEntropy
firstorder_InterquartileRange
glszm_SmallAreaEmphasis
firstorder_Range
glrlm_ShortRunEmphasis
glcm_SumSquares
glrlm_RunLengthNonUniformityNormalized
glszm_SizeZoneNonUniformity
ngtdm_Busyness
glrlm_RunPercentage
glcm_ClusterTendency
gldm_DependenceNonUniformityNormalized
glrlm_LongRunEmphasis
firstorder_Kurtosis
glcm_JointEnergy
firstorder_Energy
firstorder_TotalEnergy
gldm_LargeDependenceHighGrayLevelEmphasis
glrlm_RunVariance
glcm_MaximumProbability
gldm_LargeDependenceEmphasis
glcm_Imc2
glszm_ZoneEntropy
firstorder_Minimum
firstorder_90Percentile
glcm_ClusterProminence
ngtdm_Contrast
gldm_SmallDependenceHighGrayLevelEmphasis
gldm_DependenceEntropy
glcm_JointAverage
gldm_DependenceVariance
glszm_SmallAreaHighGrayLevelEmphasis
glrlm_LongRunHighGrayLevelEmphasis
ngtdm_Strength
glszm_HighGrayLevelZoneEmphasis
glrlm_HighGrayLevelRunEmphasis
gldm_HighGrayLevelEmphasis
glrlm_ShortRunHighGrayLevelEmphasis
glcm_Imc1
glcm_Idn
glcm_Autocorrelation
gldm_LargeDependenceLowGrayLevelEmphasis
ngtdm_Coarseness
firstorder_Mean
glcm_Correlation
firstorder_Median
glcm_Idmn
glszm_LowGrayLevelZoneEmphasis
glszm_SmallAreaLowGrayLevelEmphasis
glrlm_LongRunLowGrayLevelEmphasis
gldm_SmallDependenceLowGrayLevelEmphasis
glrlm_LowGrayLevelRunEmphasis
glrlm_ShortRunLowGrayLevelEmphasis
gldm_LowGrayLevelEmphasis
firstorder_Skewness
glcm_ClusterShade
Features
0.0
0.2
0.4
0.6
0.8
1.0
ICC

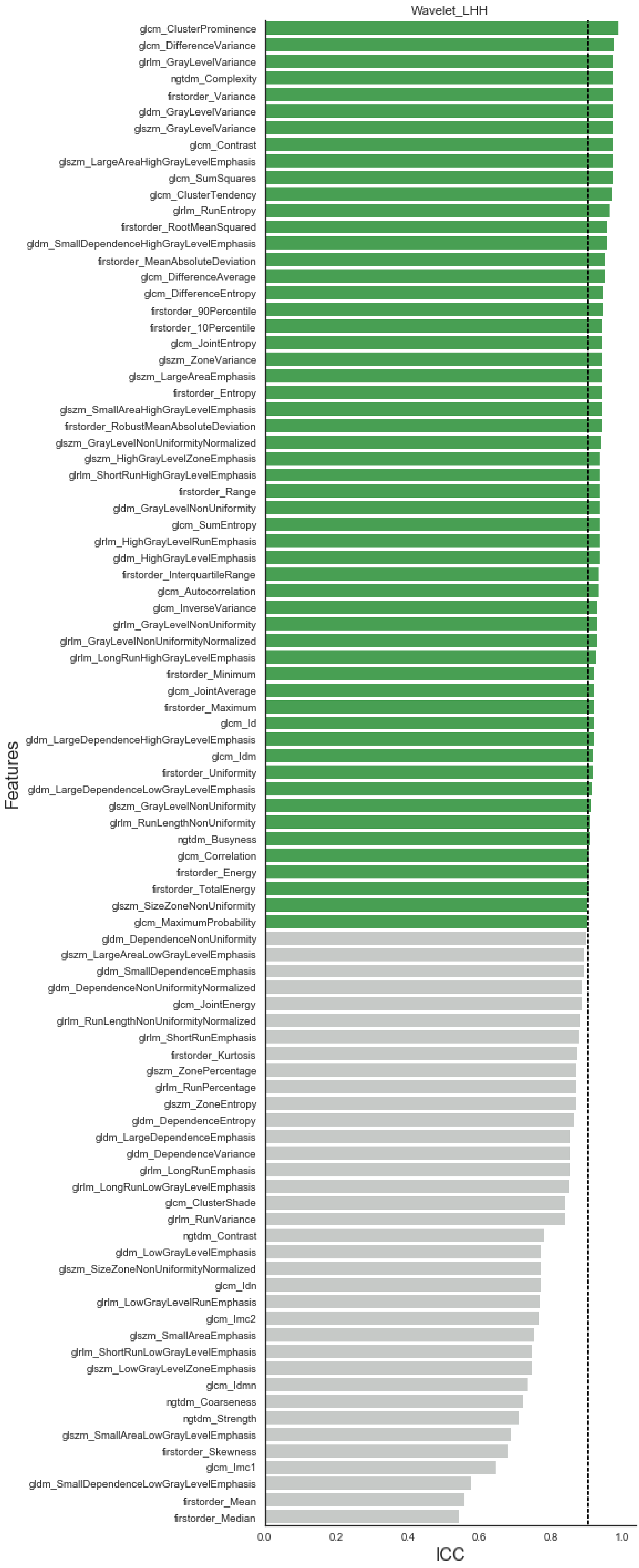
Wavelet_LHH
glcm_ClusterProminence
glcm_DifferenceVariance
glrlm_GrayLevelVariance
ngtdm_Complexity
firstorder_Variance
gldm_GrayLevelVariance
glszm_GrayLevelVariance
glcm_Contrast
glszm_LargeAreaHighGrayLevelEmphasis
glcm_SumSquares
glcm_ClusterTendency
glrlm_RunEntropy
firstorder_RootMeanSquared
gldm_SmallDependenceHighGrayLevelEmphasis
firstorder_MeanAbsoluteDeviation
glcm_DifferenceAverage
glcm_DifferenceEntropy
firstorder_90Percentile
firstorder_10Percentile
glcm_JointEntropy
glszm_ZoneVariance
glszm_LargeAreaEmphasis
firstorder_Entropy
glszm_SmallAreaHighGrayLevelEmphasis
firstorder_RobustMeanAbsoluteDeviation
glszm_GrayLevelNonUniformityNormalized
glszm_HighGrayLevelZoneEmphasis
glrlm_ShortRunHighGrayLevelEmphasis
firstorder_Range
gldm_GrayLevelNonUniformity
glcm_SumEntropy
glrlm_HighGrayLevelRunEmphasis
gldm_HighGrayLevelEmphasis
firstorder_InterquartileRange
glcm_Autocorrelation
glcm_InverseVariance
glrlm_GrayLevelNonUniformity
glrlm_GrayLevelNonUniformityNormalized
glrlm_LongRunHighGrayLevelEmphasis
firstorder_Minimum
glcm_JointAverage
firstorder_Maximum
glcm_Id
gldm_LargeDependenceHighGrayLevelEmphasis
glcm_Idm
firstorder_Uniformity
gldm_LargeDependenceLowGrayLevelEmphasis
glszm_GrayLevelNonUniformity
glrlm_RunLengthNonUniformity
ngtdm_Busyness
glcm_Correlation
firstorder_Energy
firstorder_TotalEnergy
glszm_SizeZoneNonUniformity
glcm_MaximumProbability
gldm_DependenceNonUniformity
glszm_LargeAreaLowGrayLevelEmphasis
gldm_SmallDependenceEmphasis
gldm_DependenceNonUniformityNormalized
glcm_JointEnergy
glrlm_RunLengthNonUniformityNormalized
glrlm_ShortRunEmphasis
firstorder_Kurtosis
glszm_ZonePercentage
glrlm_RunPercentage
glszm_ZoneEntropy
gldm_DependenceEntropy
gldm_LargeDependenceEmphasis
gldm_DependenceVariance
glrlm_LongRunEmphasis
glrlm_LongRunLowGrayLevelEmphasis
glcm_ClusterShade
glrlm_RunVariance
ngtdm_Contrast
gldm_LowGrayLevelEmphasis
glszm_SizeZoneNonUniformityNormalized
glcm_Idn
glrlm_LowGrayLevelRunEmphasis
glcm_Imc2
glszm_SmallAreaEmphasis
glrlm_ShortRunLowGrayLevelEmphasis
glszm_LowGrayLevelZoneEmphasis
glcm_Idmn
ngtdm_Coarseness
ngtdm_Strength
glszm_SmallAreaLowGrayLevelEmphasis
firstorder_Skewness
glcm_Imc1
gldm_SmallDependenceLowGrayLevelEmphasis
firstorder_Mean
firstorder_Median
Features
0.0
0.2
0.4
0.6
0.8
1.0
ICC

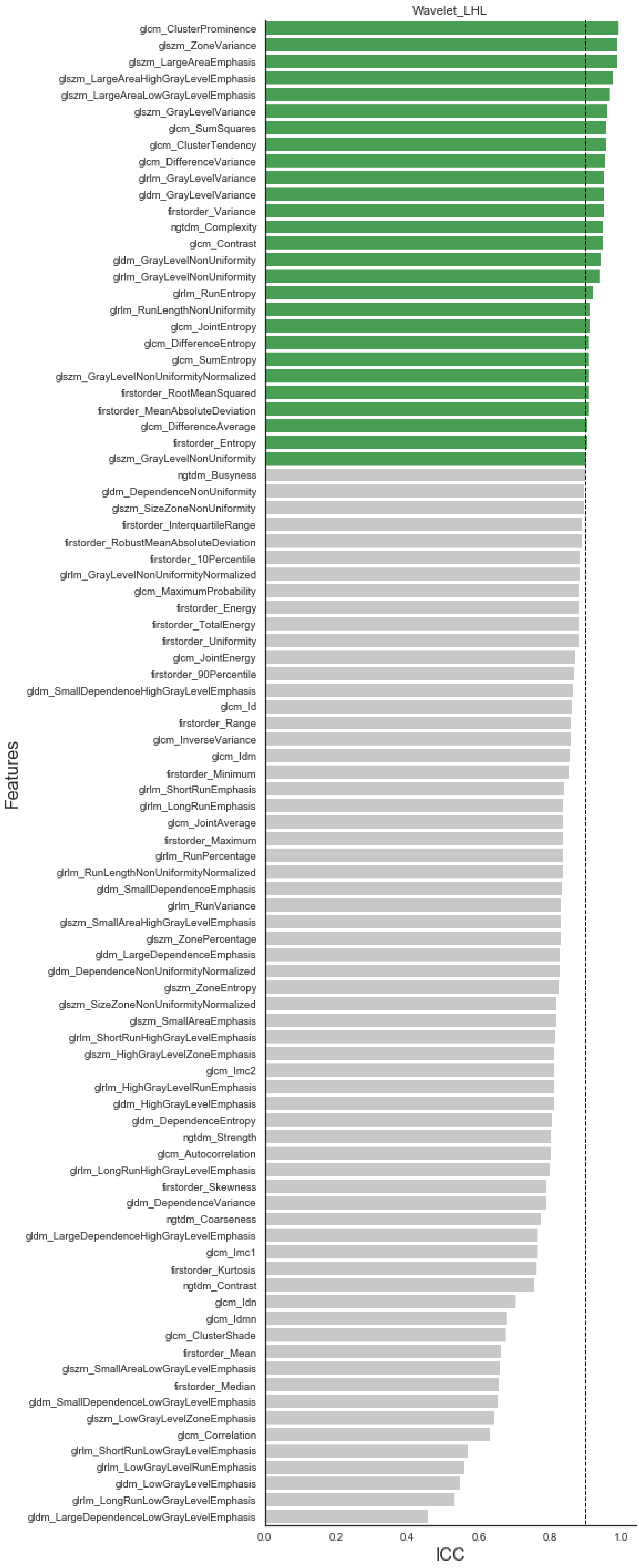
Wavelet_LHL
glcm_ClusterProminence
glszm_ZoneVariance
glszm_LargeAreaEmphasis
glszm_LargeAreaHighGrayLevelEmphasis
glszm_LargeAreaLowGrayLevelEmphasis
glszm_GrayLevelVariance
glcm_SumSquares
glcm_ClusterTendency
glcm_DifferenceVariance
glrlm_GrayLevelVariance
gldm_GrayLevelVariance
firstorder_Variance
ngtdm_Complexity
glcm_Contrast
gldm_GrayLevelNonUniformity
glrlm_GrayLevelNonUniformity
glrlm_RunEntropy
glrlm_RunLengthNonUniformity
glcm_JointEntropy
glcm_DifferenceEntropy
glcm_SumEntropy
glszm_GrayLevelNonUniformityNormalized
firstorder_RootMeanSquared
firstorder_MeanAbsoluteDeviation
glcm_DifferenceAverage
firstorder_Entropy
glszm_GrayLevelNonUniformity
ngtdm_Busyness
gldm_DependenceNonUniformity
glszm_SizeZoneNonUniformity
firstorder_InterquartileRange
firstorder_RobustMeanAbsoluteDeviation
firstorder_10Percentile
glrlm_GrayLevelNonUniformityNormalized
glcm_MaximumProbability
firstorder_Energy
firstorder_TotalEnergy
firstorder_Uniformity
glcm_JointEnergy
firstorder_90Percentile
gldm_SmallDependenceHighGrayLevelEmphasis
glcm_Id
firstorder_Range
glcm_InverseVariance
glcm_Idm
firstorder_Minimum
glrlm_ShortRunEmphasis
glrlm_LongRunEmphasis
glcm_JointAverage
firstorder_Maximum
glrlm_RunPercentage
glrlm_RunLengthNonUniformityNormalized
gldm_SmallDependenceEmphasis
glrlm_RunVariance
glszm_SmallAreaHighGrayLevelEmphasis
glszm_ZonePercentage
gldm_LargeDependenceEmphasis
gldm_DependenceNonUniformityNormalized
glszm_ZoneEntropy
glszm_SizeZoneNonUniformityNormalized
glszm_SmallAreaEmphasis
glrlm_ShortRunHighGrayLevelEmphasis
glszm_HighGrayLevelZoneEmphasis
glcm_Imc2
glrlm_HighGrayLevelRunEmphasis
gldm_HighGrayLevelEmphasis
gldm_DependenceEntropy
ngtdm_Strength
glcm_Autocorrelation
glrlm_LongRunHighGrayLevelEmphasis
firstorder_Skewness
gldm_DependenceVariance
ngtdm_Coarseness
gldm_LargeDependenceHighGrayLevelEmphasis
glcm_Imc1
firstorder_Kurtosis
ngtdm_Contrast
glcm_Idn
glcm_Idmn
glcm_ClusterShade
firstorder_Mean
glszm_SmallAreaLowGrayLevelEmphasis
firstorder_Median
gldm_SmallDependenceLowGrayLevelEmphasis
glszm_LowGrayLevelZoneEmphasis
glcm_Correlation
glrlm_ShortRunLowGrayLevelEmphasis
glrlm_LowGrayLevelRunEmphasis
gldm_LowGrayLevelEmphasis
glrlm_LongRunLowGrayLevelEmphasis
gldm_LargeDependenceLowGrayLevelEmphasis
Features
0.0
0.2
0.4
0.6
0.8
1.0
ICC

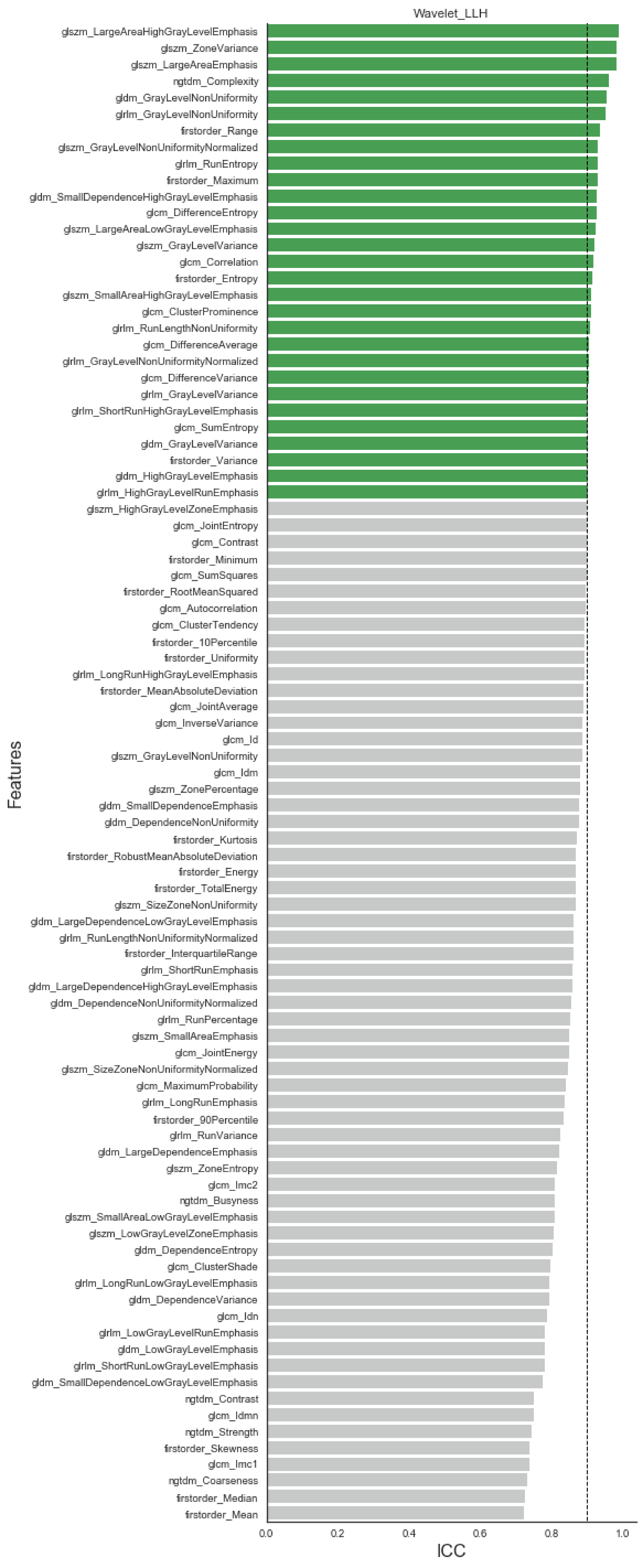

Wavelet_LLH
glszm_LargeAreaHighGrayLevelEmphasis
glszm_ZoneVariance
glszm_LargeAreaEmphasis
ngtdm_Complexity
gldm_GrayLevelNonUniformity
glrlm_GrayLevelNonUniformity
firstorder_Range
glszm_GrayLevelNonUniformityNormalized
glrlm_RunEntropy
firstorder_Maximum
gldm_SmallDependenceHighGrayLevelEmphasis
glcm_DifferenceEntropy
glszm_LargeAreaLowGrayLevelEmphasis
glszm_GrayLevelVariance
glcm_Correlation
firstorder_Entropy
glszm_SmallAreaHighGrayLevelEmphasis
glcm_ClusterProminence
glrlm_RunLengthNonUniformity
glcm_DifferenceAverage
glrlm_GrayLevelNonUniformityNormalized
glcm_DifferenceVariance
glrlm_GrayLevelVariance
glrlm_ShortRunHighGrayLevelEmphasis
glcm_SumEntropy
gldm_GrayLevelVariance
firstorder_Variance
gldm_HighGrayLevelEmphasis
glrlm_HighGrayLevelRunEmphasis
glszm_HighGrayLevelZoneEmphasis
glcm_JointEntropy
glcm_Contrast
firstorder_Minimum
glcm_SumSquares
firstorder_RootMeanSquared
glcm_Autocorrelation
glcm_ClusterTendency
firstorder_10Percentile
firstorder_Uniformity
glrlm_LongRunHighGrayLevelEmphasis
firstorder_MeanAbsoluteDeviation
glcm_JointAverage
glcm_InverseVariance
glcm_Id
glszm_GrayLevelNonUniformity
glcm_Idm
glszm_ZonePercentage
gldm_SmallDependenceEmphasis
gldm_DependenceNonUniformity
firstorder_Kurtosis
firstorder_RobustMeanAbsoluteDeviation
firstorder_Energy
firstorder_TotalEnergy
glszm_SizeZoneNonUniformity
gldm_LargeDependenceLowGrayLevelEmphasis
glrlm_RunLengthNonUniformityNormalized
firstorder_InterquartileRange
glrlm_ShortRunEmphasis
gldm_LargeDependenceHighGrayLevelEmphasis
gldm_DependenceNonUniformityNormalized
glrlm_RunPercentage
glszm_SmallAreaEmphasis
glcm_JointEnergy
glszm_SizeZoneNonUniformityNormalized
glcm_MaximumProbability
glrlm_LongRunEmphasis
firstorder_90Percentile
glrlm_RunVariance
gldm_LargeDependenceEmphasis
glszm_ZoneEntropy
glcm_Imc2
ngtdm_Busyness
glszm_SmallAreaLowGrayLevelEmphasis
glszm_LowGrayLevelZoneEmphasis
gldm_DependenceEntropy
glcm_ClusterShade
glrlm_LongRunLowGrayLevelEmphasis
gldm_DependenceVariance
glcm_Idn
glrlm_LowGrayLevelRunEmphasis
gldm_LowGrayLevelEmphasis
glrlm_ShortRunLowGrayLevelEmphasis
gldm_SmallDependenceLowGrayLevelEmphasis
ngtdm_Contrast
glcm_Idmn
ngtdm_Strength
firstorder_Skewness
glcm_Imc1
ngtdm_Coarseness
firstorder_Median
firstorder_Mean
Features
0.0
0.2
0.4
0.6
0.8
1.0
ICC

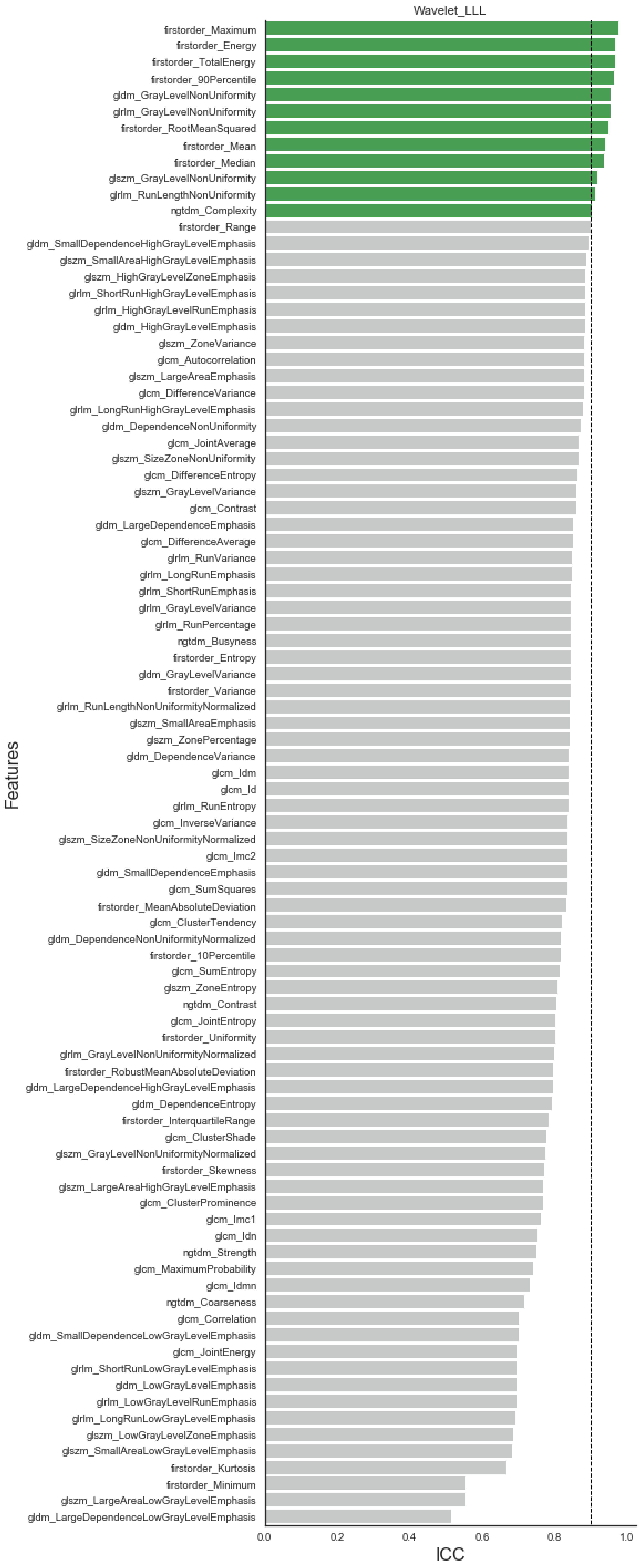

Wavelet_LLL
firstorder_Maximum
firstorder_Energy
firstorder_TotalEnergy
firstorder_90Percentile
gldm_GrayLevelNonUniformity
glrlm_GrayLevelNonUniformity
firstorder_RootMeanSquared
firstorder_Mean
firstorder_Median
glszm_GrayLevelNonUniformity
glrlm_RunLengthNonUniformity
ngtdm_Complexity
firstorder_Range
gldm_SmallDependenceHighGrayLevelEmphasis
glszm_SmallAreaHighGrayLevelEmphasis
glszm_HighGrayLevelZoneEmphasis
glrlm_ShortRunHighGrayLevelEmphasis
glrlm_HighGrayLevelRunEmphasis
gldm_HighGrayLevelEmphasis
glszm_ZoneVariance
glcm_Autocorrelation
glszm_LargeAreaEmphasis
glcm_DifferenceVariance
glrlm_LongRunHighGrayLevelEmphasis
gldm_DependenceNonUniformity
glcm_JointAverage
glszm_SizeZoneNonUniformity
glcm_DifferenceEntropy
glszm_GrayLevelVariance
glcm_Contrast
gldm_LargeDependenceEmphasis
glcm_DifferenceAverage
glrlm_RunVariance
glrlm_LongRunEmphasis
glrlm_ShortRunEmphasis
glrlm_GrayLevelVariance
glrlm_RunPercentage
ngtdm_Busyness
firstorder_Entropy
gldm_GrayLevelVariance
firstorder_Variance
glrlm_RunLengthNonUniformityNormalized
glszm_SmallAreaEmphasis
glszm_ZonePercentage
gldm_DependenceVariance
glcm_Idm
glcm_Id
glrlm_RunEntropy
glcm_InverseVariance
glszm_SizeZoneNonUniformityNormalized
glcm_Imc2
gldm_SmallDependenceEmphasis
glcm_SumSquares
firstorder_MeanAbsoluteDeviation
glcm_ClusterTendency
gldm_DependenceNonUniformityNormalized
firstorder_10Percentile
glcm_SumEntropy
glszm_ZoneEntropy
ngtdm_Contrast
glcm_JointEntropy
firstorder_Uniformity
glrlm_GrayLevelNonUniformityNormalized
firstorder_RobustMeanAbsoluteDeviation
gldm_LargeDependenceHighGrayLevelEmphasis
gldm_DependenceEntropy
firstorder_InterquartileRange
glcm_ClusterShade
glszm_GrayLevelNonUniformityNormalized
firstorder_Skewness
glszm_LargeAreaHighGrayLevelEmphasis
glcm_ClusterProminence
glcm_Imc1
glcm_Idn
ngtdm_Strength
glcm_MaximumProbability
glcm_Idmn
ngtdm_Coarseness
glcm_Correlation
gldm_SmallDependenceLowGrayLevelEmphasis
glcm_JointEnergy
glrlm_ShortRunLowGrayLevelEmphasis
gldm_LowGrayLevelEmphasis
glrlm_LowGrayLevelRunEmphasis
glrlm_LongRunLowGrayLevelEmphasis
glszm_LowGrayLevelZoneEmphasis
glszm_SmallAreaLowGrayLevelEmphasis
firstorder_Kurtosis
firstorder_Minimum
glszm_LargeAreaLowGrayLevelEmphasis
gldm_LargeDependenceLowGrayLevelEmphasis
Features
0.0
0.2
0.4
0.6
0.8
1.0
ICC

Supplementary material 3. ICC values of all unfiltered RadiomiX features with robust features (ICC > 0.9) shown in green for all 'easy tumors' (a + c) and 'challenging tumors' (b + d).

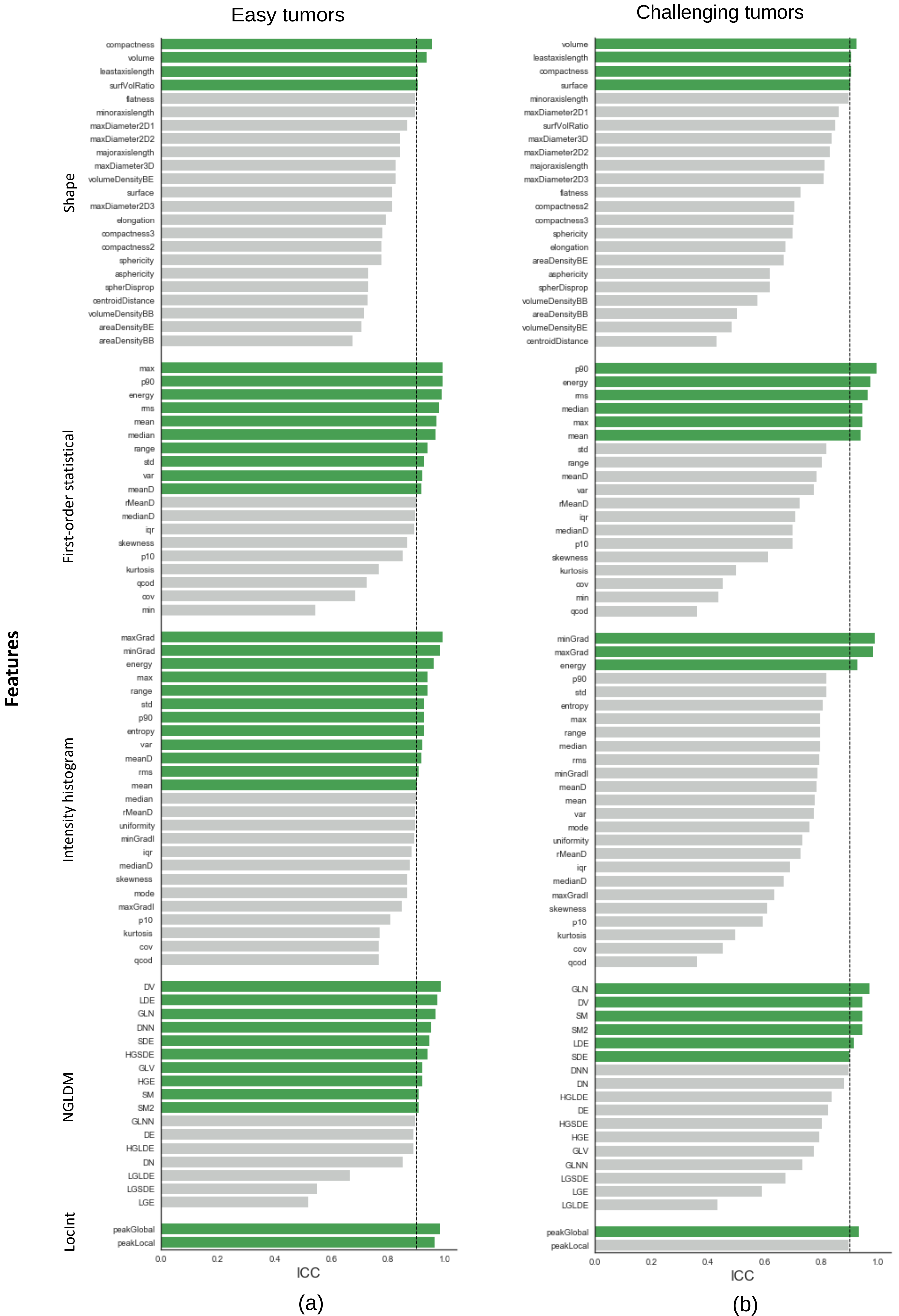

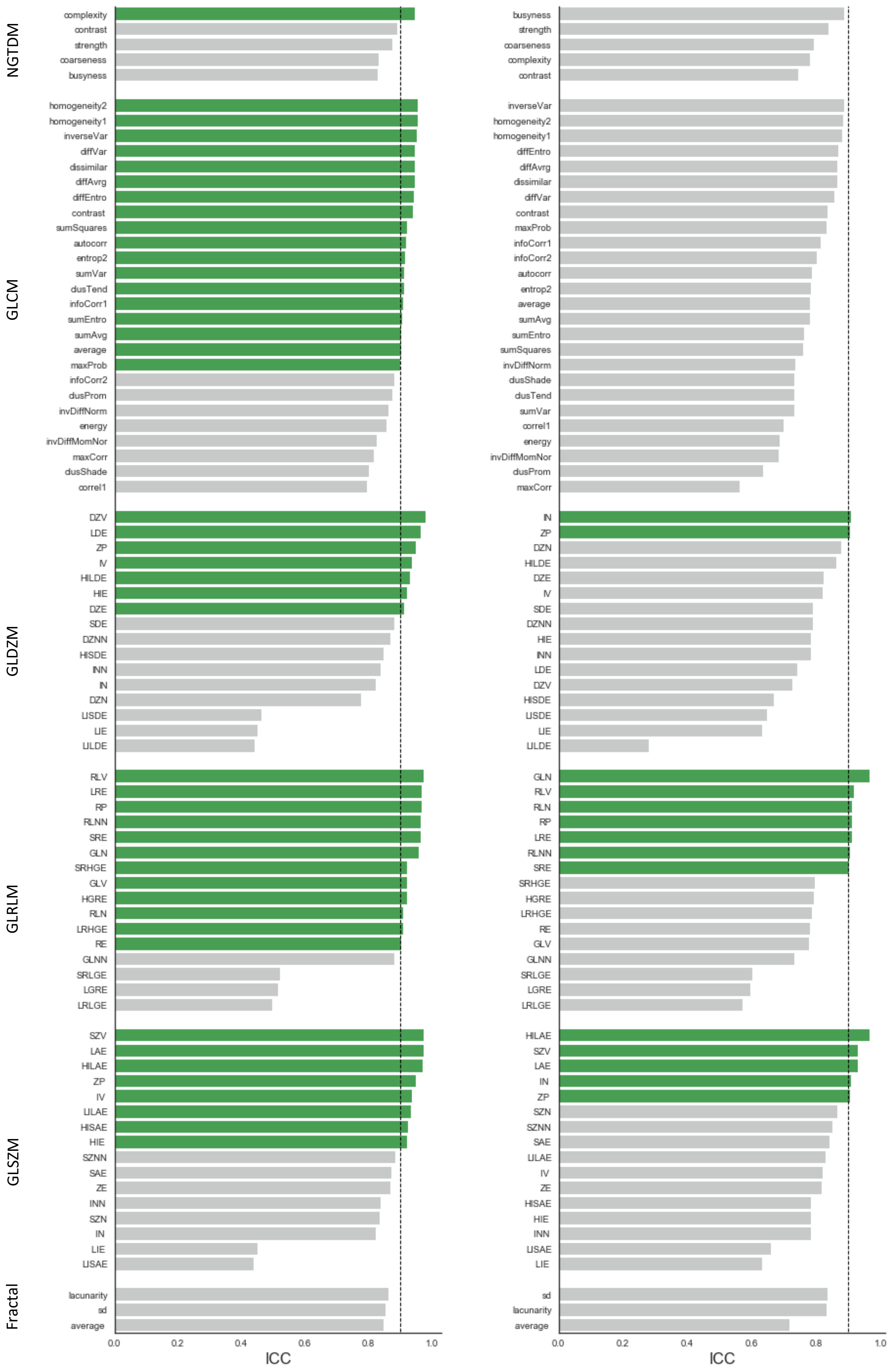
NGTDM
complexity
contrast
strength
coarseness
busyness
GLCM
homogeneity2
homogeneity1
inverseVar
diffVar
dissimilar
diffAvrg
diffEntro
contrast
sumSquares
autocorr
entrop2
sumVar
clusTend
infoCorr1
sumEntro
sumAvg
average
maxProb
infoCorr2
clusProm
invDiffNorm
energy
invDiffMomNor
maxCorr
clusShade
correl1
GLDZM
DZV
LDE
ZP
IV
HILDE
HIE
DZE
SDE
DZNN
HISDE
INN
IN
DZN
LISDE
LIE
LILDE
GLRLM
RLV
LRE
RP
RLNN
SRE
GLN
SRHGE
GLV
HGRE
RLN
LRHGE
RE
GLNN
SRLGE
LGRE
LRLGE
GLSZM
SZV
LAE
HILAE
ZP
IV
LILAE
HISAE
HIE
SZNN
SAE
ZE
INN
SZN
IN
LIE
LISAE
Fractal
lacunarity
sd
average
0.0
0.2
0.4
0.6
0.8
1.0
ICC
busyness
strength
coarseness
complexity
contrast
inverseVar
homogeneity2
homogeneity1
diffEntro
diffAvrg
dissimilar
diffVar
contrast
maxProb
infoCorr1
infoCorr2
autocorr
entrop2
average
sumAvg
sumEntro
sumSquares
invDiffNorm
clusShade
clusTend
sumVar
correl1
energy
invDiffMomNor
clusProm
maxCorr
IN
ZP
DZN
HILDE
DZE
IV
SDE
DZNN
HIE
INN
LDE
DZV
HISDE
LISDE
LIE
LILDE
GLN
RLV
RLN
RP
LRE
RLNN
SRE
SRHGE
HGRE
LRHGE
RE
GLV
GLNN
SRLGE
LGRE
LRLGE
HILAE
SZV
LAE
IN
ZP
SZN
SZNN
SAE
LILAE
IV
ZE
HISAE
HIE
INN
LISAE
LIE
sd
lacunarity
average
0.0
0.2
0.4
0.6
0.8
1.0
ICC

(c)

(d)

Supplementary material 4. ICC values of all unfiltered Pyradiomics features with robust features (ICC > 0.9) shown in green for all 'easy tumors' (a + c) and 'challenging tumors' (b + d).

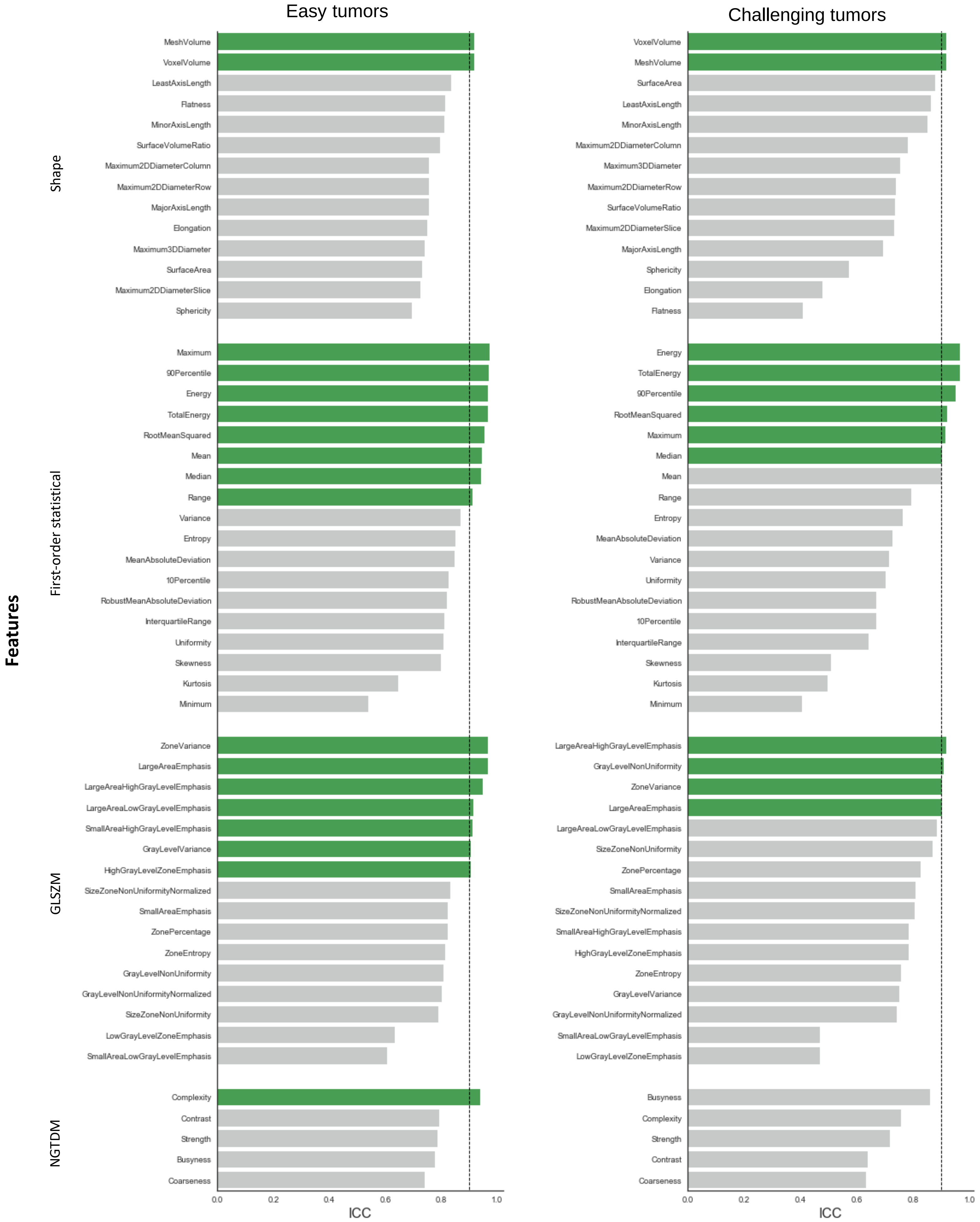

(a) (b)

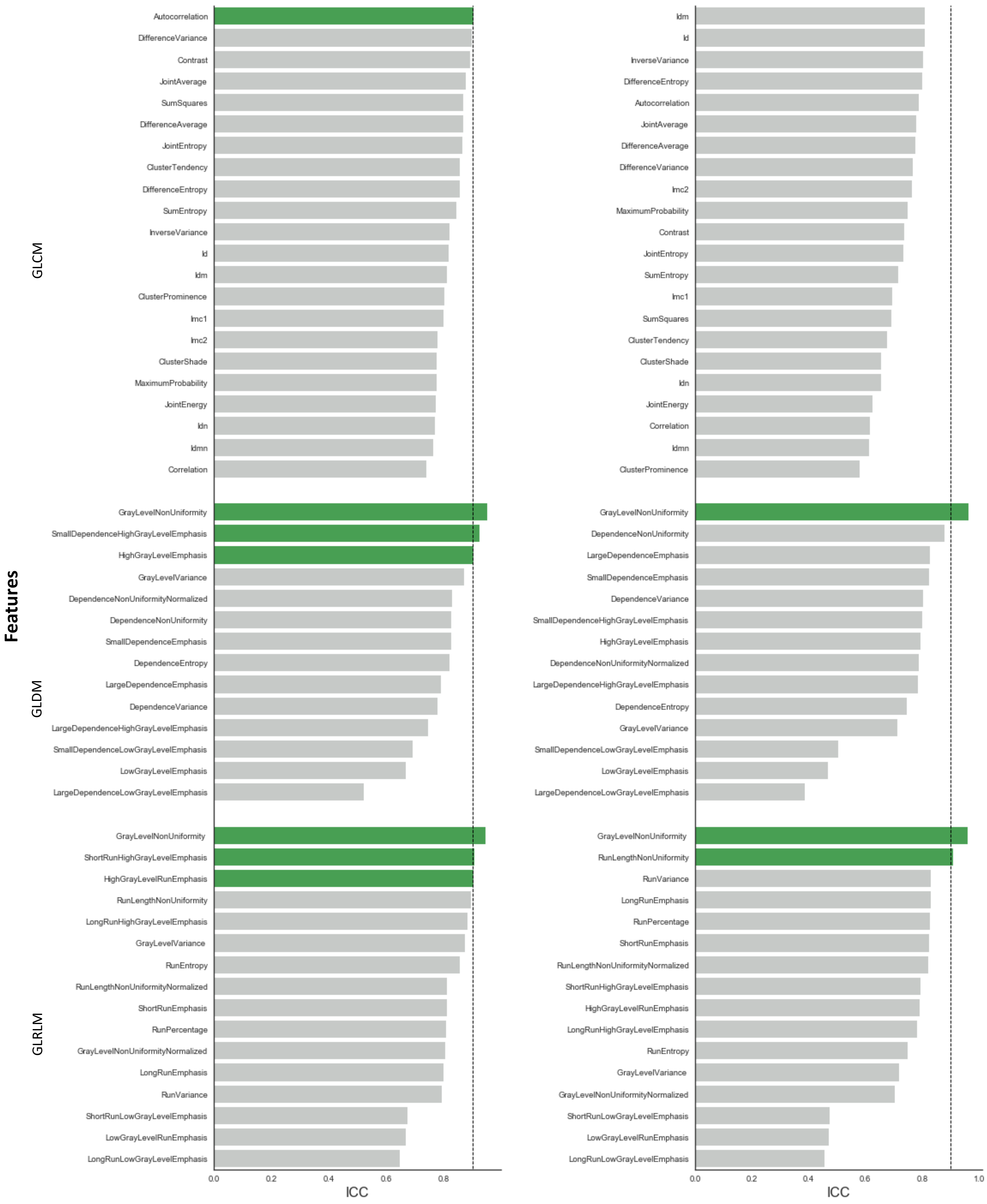
Features
GLCM
Autocorrelation
DifferenceVariance
Contrast
JointAverage
SumSquares
DifferenceAverage
JointEntropy
ClusterTendency
DifferenceEntropy
SumEntropy
InverseVariance
Id
Idm
ClusterProminence
Imc1
Imc2
ClusterShade
MaximumProbability
JointEnergy
Idn
Idmn
Correlation
GLDM
GrayLevelNonUniformity
SmallDependenceHighGrayLevelEmphasis
HighGrayLevelEmphasis
GrayLevelVariance
DependenceNonUniformityNormalized
DependenceNonUniformity
SmallDependenceEmphasis
DependenceEntropy
LargeDependenceEmphasis
DependenceVariance
LargeDependenceHighGrayLevelEmphasis
SmallDependenceLowGrayLevelEmphasis
LowGrayLevelEmphasis
LargeDependenceLowGrayLevelEmphasis
GLRLM
GrayLevelNonUniformity
ShortRunHighGrayLevelEmphasis
HighGrayLevelRunEmphasis
RunLengthNonUniformity
LongRunHighGrayLevelEmphasis
GrayLevelVariance
RunEntropy
RunLengthNonUniformityNormalized
ShortRunEmphasis
RunPercentage
GrayLevelNonUniformityNormalized
LongRunEmphasis
RunVariance
ShortRunLowGrayLevelEmphasis
LowGrayLevelRunEmphasis
LongRunLowGrayLevelEmphasis
0.0
0.2
0.4
0.6
0.8
ICC
Idm
Id
InverseVariance
DifferenceEntropy
Autocorrelation
JointAverage
DifferenceAverage
DifferenceVariance
Imc2
MaximumProbability
Contrast
JointEntropy
SumEntropy
Imc1
SumSquares
ClusterTendency
ClusterShade
Idn
JointEnergy
Correlation
Idmn
ClusterProminence
GrayLevelNonUniformity
DependenceNonUniformity
LargeDependenceEmphasis
SmallDependenceEmphasis
DependenceVariance
SmallDependenceHighGrayLevelEmphasis
HighGrayLevelEmphasis
DependenceNonUniformityNormalized
LargeDependenceHighGrayLevelEmphasis
DependenceEntropy
GrayLevelVariance
SmallDependenceLowGrayLevelEmphasis
LowGrayLevelEmphasis
LargeDependenceLowGrayLevelEmphasis
GrayLevelNonUniformity
RunLengthNonUniformity
RunVariance
LongRunEmphasis
RunPercentage
ShortRunEmphasis
RunLengthNonUniformityNormalized
ShortRunHighGrayLevelEmphasis
HighGrayLevelRunEmphasis
LongRunHighGrayLevelEmphasis
RunEntropy
GrayLevelVariance
GrayLevelNonUniformityNormalized
ShortRunLowGrayLevelEmphasis
LowGrayLevelRunEmphasis
LongRunLowGrayLevelEmphasis
0.0
0.2
0.4
0.6
0.8
1.0
ICC

(c)

(d)

Supplementary material 5. Feature description of RadiomiX software.

Feature descriptions

* - Not described in IBSI feature definitions

*Fractal Dimension features**

The Fractal Dimension (FD) of the image is computed as described by Al-Kadi and Watson (1). Given the FD processed image I, with N elements:

1. Average

$$average = \frac{1}{N}\sum I$$

2. Lacunarity

$$lacunarity = \frac{\frac{1}{N}\sum I^2}{\frac{1}{N^2}\sum I} - 1$$

3. Standard deviation

$$standard\ deviation = \left(\frac{1}{N-1}\sum (I-\bar{I})^2\right)^{1/2}$$

Where $\bar{I}$ is the mean of I.

First-order Grey-level statistics

First-order Grey-level statistics describe the distribution of grey values within the volume. Let X denote the three dimensional image matrix with N voxels, P the first order histogram, $P(i)$ the fraction of voxels with intensity level i and N_l the number of discrete intensity levels.

1. Energy

$$energy = \sum_{i=1}^{N} X(i)^2$$

Energy is also known as the sum of squares.

2. Entropy

$$entropy = \sum_{i=1}^{N_l} P(i)\log_2 P(i)$$

Note: Defined by IBSI as Intensity Histogram Entropy.

3. Kurtosis

$$kurtosis = \frac{\frac{1}{N}\sum_{i=1}^{N}(X(i)-\bar{X})^4}{\left(\frac{1}{N}\sum_{i=1}^{N}(X(i)-\bar{X})^2\right)^2}$$

where $\bar{X}$ is the mean of X.
Note: The IBSI feature definition implements excess kurtosis, where kurtosis is corrected by -3, yielding 0 for normal distributions. The kurtosis presented above is not corrected, yielding a value 3 higher than the IBSI kurtosis.

4. Maximum

The maximum grey value of X.

$$maximum = \max(X)$$

5. Mean

The mean grey value of X.

$$mean = \frac{1}{N}\sum_{i=1}^{N} X(i)$$

Mean ADC values of the entire cohort was calculated over the mean of each lesion

6. Mean absolute deviation

The mean of the absolute deviations of all voxel intensities around the mean intensity value.

$$mean\ absolute\ deviation = \frac{1}{N}\sum_{i=1}^{N}\left|X(i) - \bar{X}\right|$$

where $\bar{X}$ is the mean of X.

7. Median

The sample median of X, or the 50th percentile of X.
Median ADC values of the entire cohort was calculated over the mean of each lesion

8. Minimum

The minimum intensity value of X.

$$minimum = \min(X)$$

9. Range

The range of intensity values of X.

$$range = \max(X) - \min(X)$$

10. Root mean square (RMS)

The quadratic mean, or the square root of the mean of squares of all voxel intensities.

$$RMS = \sqrt{\frac{\sum_{i}^{N} X(i)^2}{N}}$$

11. Skewness

$$skewness = \frac{\frac{1}{N}\sum_{i=1}^{N}(X(i) - \bar{X})^3}{\left(\sqrt{\frac{1}{N}\sum_{i=1}^{N}(X(i) - \bar{X})^2}\right)^3}$$

where $\bar{X}$ is the mean of X.

12. Standard deviation *

$$standard\ deviation = \left(\frac{1}{N-1}\sum_{i=1}^{N}(X(i)-\bar{X})^2\right)^{1/2}$$

where $\bar{X}$ is the mean of X.

13. Robust mean absolute deviation

The mean absolute deviation (0) of only those voxels in X with a grey value between the 10th and 90th percentile.

14. 10th percentile
The 10th percentile of X, a robust alternative to the minimum grey value (8).

15. 90th percentile
The 90th percentile of X, a robust alternative to the maximum grey value (4).

16. Interquartile range
The interquartile range is defined as the 75th minus the 25th percentile of X.

17. Uniformity

$$uniformity = \sum_{i=1}^{N_l} P(i)^2$$

Note: Defined by IBSI as Intensity Histogram Uniformity.

18. Variance

$$variance = \frac{1}{N-1}\sum_{i=1}^{N}(X(i)-\bar{X})^2$$

where $\bar{X}$ is the mean of X. Variance is the square of the standard deviation (12).

Intensity histogram features

Intensity histogram features describe the distribution of grey values within the volume, after discretization into intensity level bins was applied. Let:

$X_d = \{X_{d,1}, X_{d,2}, \dots, X_{d,N_v}\}$ be the set of discretized intensity values of the N_v voxels in the volume of interest,
$H = \{n_1, n_2, \dots\}$ be the histogram with frequency count n_i of each discretized intensity level i in X_d,
N_g be the number of discretized intensity values (bins) in the volume of interest,
p_i be the occurrence probability for each bin i of the histogram N_g, where $p_i = n_i/N_v$.

1. Coefficient of variance (cov)

$$cov = \frac{\text{standard deviation}}{\text{mean}}$$

2. Energy

$$energy = \sum_{j=1}^{N_v} X_d(j)^2$$

Energy is also known as the sum of squares.

3. Entropy

$$entropy = -\sum_{i=1}^{N_g} P(i)\log_2 P(i)$$

4. Interquartile range (iqr)

$$IQR = P_{75} - P_{25}$$

where P_{25} and P_{75} are the 25th and 75th percentile of X_d, respectively.

5. Kurtosis

$$kurtosis = \frac{\frac{1}{N_v}\sum_{j=1}^{N_v}(X_d(j) - \bar{X}_d)^4}{\left(\frac{1}{N_v}\sum_{j=1}^{N_v}(X_d(j) - \bar{X}_d)^2\right)^2}$$

where $\bar{X}_d$ is the mean of X_d.

6. Maximum

The maximum discretized intensity value of X_d.

$$maximum = \max(X_d)$$

7. Maximum histogram gradient (maxgrad)

$$maxgrad = \max(H')$$

Where H' is the histogram gradient, defined as:

$$H' = \{H(2) - H(1), \dots, \frac{H(i+1) - H(i-1)}{2}, \dots, H(N_g) - H(N_g - 1)\}$$

8. Maximum histogram gradient intensity level (maxgradi)

The discretized intensity level i corresponding to the maximum histogram gradient.

9. Mean

The mean discretized intensity value of X_d.

$$mean = \frac{1}{N_v}\sum_{j=1}^{N_v} X_d(j)$$

10. Mean absolute deviation (meand)

The mean of the absolute deviations of all discretized intensity levels around the mean of X_d.

$$meand = \frac{1}{N_v}\sum_{j=1}^{N_v} |X_d(j) - \overline{X_d}|$$

where $\bar{X}_d$ is the mean of X_d.

11. Median

The sample median of X_d or the 50th percentile of X_d.

12. Median absolute deviation (mediand)

The dispersion from the median of X_d.

$$mediand = \frac{1}{N_v}\sum_{j=1}^{N_v}|X_d(j) - M|$$

where M is the median of X_d.

13. Minimum

The minimum discretized intensity value of X_d.

$$minimum = \min(X_d)$$

14. Minimum histogram gradient (mingrad)

$$mingrad = \min(H')$$

Where H' is the histogram gradient, defined as:

$$H' = \{H(2) - H(1), \ldots, \frac{H(i+1) - H(i-1)}{2}, \ldots, H(N_g) - H(N_g - 1)\}$$

15. Minimum histogram gradient intensity level (mingradi)

The discretized intensity level i corresponding to the minimum histogram gradient.

16. Mode

The mode of X_d is the most frequently occurring discretized image level present. In case multiple bins have the highest count n_i, the mode is the smallest of those values.

17. Uniformity

$$uniformity = \sum_{i=1}^{N_g} P(i)^2$$

Note: Defined by IBSI as Intensity Histogram Uniformity.

18. Range

The range of bins in the histogram, i.e. the width of the histogram.

$$range = \max(X_d) - \min(X_d)$$

19. Root mean square (RMS):

$$RMS = \sqrt{\frac{\sum_{j=1}^{N_v} X_d(j)^2}{N_v}}$$

20. Robust mean absolute deviation (rmeand)

Similar to mean absolute deviation, but in this case only considering the set of intensity levels in the range between the 10th and 90th percentile of X_d.

$$rmeand = \frac{1}{N_{10-90}}\sum_{j=1}^{N_{10-90}}\left|X_{d,10-90}(j) - \bar{X}_{d,10-90}\right|$$

where X_{10-90} represents the set of N_{10-90} voxels in X_d whose discretized intensity levels fall within the range of the 10th till the 90th percentile of X_d.

21. Skewness

$$skewness = \frac{\frac{1}{N_v}\sum_{j=1}^{N_v}(X_d(j) - \bar{X}_d)^3}{\left(\sqrt{\frac{1}{N_v}\sum_{j=1}^{N_v}(X_d(j) - \bar{X}_d)^2}\right)^3}$$

22. Standard deviation

$$standard\ deviation = \left(\frac{1}{N_v - 1}\sum_{j=1}^{N_v}(X_d(j) - \bar{X}_d)^2\right)^{1/2}$$

23. Variance

The variance of X_d.

$$variance = \frac{1}{N_v - 1}\sum_{j=1}^{N_v}(X_d(j) - \overline{X_d})^2$$

where $\overline{X_d}$ is the mean of X_d.

24. 10th percentile

The 10th percentile of X_d.

25. 90th percentile

The 90th percentile of X_d.

26. Quartile coefficient of dispersion (qcod)

The quartile coefficient of dispersion is a robust alternative to the coefficient of variance.

$$qcod = \frac{P_{75} - P_{25}}{P_{75} + P_{25}}$$

where P_{25} and P_{75} are the 25th and 75th percentile of X_d, respectively.

*Local Intensity features **

Local Intensity (LocInt) features are defined based on local intensity values around a center voxel (2).

1. Local intensity peak

Mean intensity level in a 1 cm^3 spherical volume, centered on the voxel with the maximum intensity level in the volume of interest. In case multiple voxels contain the maximum intensity level, the highest mean intensity level of all spherical volumes is used.

2. Global Intensity peak

Similar to local intensity peak, but in this case the mean intensity level in a 1 cm^3 spherical volume is calculated for every voxel in the volume of interest. The highest mean intensity level of all spherical volumes is selected as the global intensity peak feature.

Geometric features

Geometric features describe the shape and size of the volume of interest. Let V be the volume and A the surface area of the volume of interest. Let N be the total number of voxels, $X = \{\vec{X}_1, \vec{X}_2, \ldots, \vec{X}_N\}$ the set of N Cartesian coordinate vectors and $I = \{I_1, I_2, \ldots, I_N\}$ the corresponding intensity values.

1. Asphericity

$$asphericity = \left(\frac{1}{36\pi}\frac{A^3}{V^2}\right)^{\frac{1}{3}} - 1$$

2. Centroid distance

The centroid distance is the Euclidean distance between the geometric centroid (C_g) and the centroid weighing each voxel by its intensity value (C_i). The centroid distance is a measure of how close the high intensity values are to the geometric center.

$$C_g = \frac{1}{N}\sum_{i=1}^{N} \vec{X}_i$$

$$C_i = \frac{\sum_{i=1}^{N} I\vec{X}_i}{\sum_{i=1}^{N} I}$$

$$centroid\ distance = \left\|C_g - C_i\right\|$$

3. Compactness 1

Compactness is a measure of how much the volume resembles a sphere, as described by Aerts et al. (3).

$$compactness\ 1 = \frac{V}{\sqrt{\pi}A^{\frac{2}{3}}}$$

4. Compactness 2

$$compactness\ 2 = 36\pi\frac{V^2}{A^3}$$

5. Compactness 3

$$compactness\ 3 = \frac{V}{\sqrt{\pi}A^{\frac{3}{2}}}$$

A dimensionless alternative to Compactness (3), as described by Aerts et al. (3).

6. Maximum diameter

The maximum diameter is the largest pairwise difference between voxels on the surface of the volume, in 3D and for each plane separately. The following diameters are calculated:

a. The maximum three-dimensional tumor diameter.
b. The maximum two-dimensional diameter of all transversal planes.
c. The maximum two-dimensional diameter of all sagittal planes.
d. The maximum two-dimensional diameter of all coronal planes.

7. Major axis length

Axis lengths are measures of the extent of the volume along its three principle axis. Principle component analysis (PCA) on the x, y and z coordinates of all voxels within the volume is used to determine the three orthogonal eigenvectors and corresponding eigenvalues ($\lambda_{max}, \lambda_{minor}, \lambda_{min}$). The major axis length is the largest eigenvalue (λ_{max}) as determined by PCA.

8. Minor axis length

The largest eigenvalue (λ_{minor}) as determined by PCA.

9. Least axis length

The smallest eigenvalue (λ_{min}) as determined by PCA.

10. Elongation

$$elongation = \frac{\lambda_{minor}}{\lambda_{max}}$$

11. Flatness

$$flatness = \frac{\lambda_{min}}{\lambda_{max}}$$

12. Spherical disproportion (4)

Spherical disproportion is a measure of how much the volume resembles a sphere.

$$spherical\ disproportion = \frac{A}{4\pi R^2}$$

Where A is the surface area and R is the radius of a sphere with the same volume as the tumor, obtained through:

$$R = \sqrt[3]{\frac{3V}{4\pi}}$$

13. Sphericity (4)

Sphericity is a measure of how much the volume resembles a sphere.

$$sphericity = \frac{\pi^{\frac{1}{3}}(6V)^{\frac{2}{3}}}{A} = \frac{(36\pi V^2)^{\frac{1}{3}}}{A}$$

14. Surface area

The surface area is calculated by triangulation (i.e. dividing the surface into connected triangles, which define the isosurface enclosing the volume) and is defined as:

$$surface\ area = \sum_{i=1}^{N} \frac{1}{2} |a_i b_i \times a_i c_i|$$

Where N is the total number of triangles covering the surface and a, b and c are edge vectors of the triangles.

15. Surface to volume ratio

$$surface\ to\ volume\ ratio = \frac{A}{V}$$

16. Volume

The volume is defined as the number of voxels within the volume multiplied by the voxel volume.

$$volume = Nv$$

Where v is the volume of a single voxel.

Note: In the IBSI feature definitions, a more precise approximation of the volume is used. That method uses tetrahedrons consisting of the origin and faces in the ROI. Although the method implemented here overestimates the volume, especially in small volumes, the difference will be negligible in large ROIs.

Grey-Level Co-Occurrence Matrix based features

Grey-level co-occurrence matrix (GLCM) based features, as originally described by Haralick et al (5). A normalized GLCM is defined as $P(i,j;\delta,\alpha)$, a matrix with size $N_g \times N_g$ describing the second-order joint probability function of an image, where the (i,j)th element represents the number of times the combination of intensity levels i and j occur in two pixels in the image, that are separated by a distance of δ pixels in direction α, and N_g is the maximum discrete intensity level in the image. Let:

$P(i,j)$ be the normalized (i.e. $\sum P(i,j) = 1$) co-occurrence matrix, generalized for any δ and α ,

$p_x(i) = \sum_{j=1}^{N_g} P(i,j)$,

$p_y(j) = \sum_{i=1}^{N_g} P(i,j)$,

μ_x be the mean of p_x, where $\mu_x = \sum_{i=1}^{N_g} \sum_{j=1}^{N_g} iP(i,j)$

μ_y be the mean of p_y, where $\mu_y = \sum_{i=1}^{N_g} \sum_{j=1}^{N_g} jP(i,j)$

σ_x be the standard deviation of p_x, where $\sigma_x^2 = \sum_{i=1}^{N_g} \sum_{j=1}^{N_g} P(i,j)(i-\mu_x)^2$

σ_y be the standard deviation of p_y, where $\sigma_y^2 = \sum_{i=1}^{N_g} \sum_{j=1}^{N_g} P(i,j)(j-\mu_y)^2$

$p_{x+y}(k) = \sum_{i=1}^{N_g} \sum_{j=1}^{N_g} P(i,j),\ i+j=k,\ k=2,3,\dots,2N_g,$

$p_{x-y}(k) = \sum_{i=1}^{N_g} \sum_{j=1}^{N_g} P(i,j),\ |i-j|=k,\ k=0,1,\dots,N_g-1,$

$HXY1 = -\sum_{i=1}^{N_g} \sum_{j=1}^{N_g} P(i,j)\ln(p_x(i)p_y(j))$,

$HXY2 = -\sum_{i=1}^{N_g} \sum_{j=1}^{N_g} p_x(i)p_y(j)\ln(p_x(i)p_y(j))$.

$HX = -\sum p_x \ln(p_x)$

$HY = -\sum p_y \ln(p_y)$

1. Average (μ)

$$average\ (\mu) = \frac{\sum_{i=1}^{N_g} \sum_{j=1}^{N_g} (i+j)P(i,j)}{2}$$

Note that for a symmetrical GLCM, $\mu = \mu_x = \mu_y$.

2. Autocorrelation

$$autocorrelation = \sum_{i=1}^{N_g} \sum_{j=1}^{N_g} ijP(i,j)$$

3. Cluster Prominence

$$cluster\ prominence = \sum_{i=1}^{N_g}\sum_{j=1}^{N_g}\left[i + j - \mu_x - \mu_y\right]^4 P(i,j)$$

4. Cluster Shade

$$cluster\ shade = \sum_{i=1}^{N_g}\sum_{j=1}^{N_g}\left[i + j - \mu_x - \mu_y\right]^3 P(i,j)$$

5. Cluster Tendency

$$cluster\ tendency = \sum_{i=1}^{N_g}\sum_{j=1}^{N_g}\left[i + j - \mu_x - \mu_y\right]^2 P(i,j)$$

6. Contrast (6)

$$contrast = \sum_{i=1}^{N_g}\sum_{j=1}^{N_g}|i - j|^2 P(i,j) = \sum_{k=0}^{N_g-1} k^2\, p_{x-y}(k)$$

7. Correlation

$$correlation = \frac{\sum_{i=1}^{N_g}\sum_{j=1}^{N_g} ijP(i,j) - \mu_x\mu_y}{\sigma_x\sigma_y}$$

8. Difference Average (μ_{x-y})

$$difference\ average\ (\mu_{x-y}) = \sum_{k=0}^{N_g-1} kp_{x-y}$$

9. Difference Entropy

$$difference\ entropy = -\sum_{i=0}^{N_g-1} P_{x-y}(i)\log_2\left[P_{x-y}(i)\right]$$

10. Difference Variance

$$difference\ variance = \sum_{i=0}^{N_g-1} (i - \mu_{x-y})^2 P_{x-y}(i)$$

11. Dissimilarity

$$dissimilarity = \sum_{i=1}^{N_g}\sum_{j=1}^{N_g}|i - j| P(i,j)$$

12. Energy (7)

$$energy = \sum_{i=1}^{N_g}\sum_{j=1}^{N_g}[P(i,j)]^2$$

This feature is also called Angular Second Moment (ASM) and Uniformity (6).

13. Entropy (H)

$$entropy\ (H) = -\sum_{i=1}^{N_g}\sum_{j=1}^{N_g}P(i,j)\log_2[P(i,j)]$$

14. Homogeneity 1

$$homogeneity\ 1 = \sum_{i=1}^{N_g}\sum_{j=1}^{N_g}\frac{P(i,j)}{1+|i-j|}$$

This feature is also called Inverse Difference (6).

15. Homogeneity 2 (7)

$$homogeneity\ 2 = \sum_{i=1}^{N_g}\sum_{j=1}^{N_g}\frac{P(i,j)}{1+|i-j|^2}$$

This feature is also called Inverse Difference Moment (6).

16. Informational measure of correlation 1 (IMC1)

$$IMC1 = \frac{H - HXY1}{\max\{HX, HY\}}$$

Where H is the entropy (13).

17. Informational measure of correlation 2 (IMC2)

$$IMC2 = \sqrt{1 - e^{-2(HXY2-H)}}$$

Where H is the entropy (13).

18. Inverse Difference Moment Normalized (IDMN)

$$IDMN = \sum_{i=1}^{N_g}\sum_{j=1}^{N_g}\frac{P(i,j)}{1+\left(\frac{|i-j|^2}{N_g^2}\right)}$$

19. Inverse Difference Normalized (IDN)

$$IDN = \sum_{i=1}^{N_g}\sum_{j=1}^{N_g}\frac{P(i,j)}{1+\left(\frac{|i-j|}{N_g}\right)}$$

20. Inverse variance

$$inverse\ variance = \sum_{i=1}^{N_g}\sum_{j=1}^{N_g}\frac{P(i,j)}{|i-j|^2} \quad ,i \neq j$$

21. Maximal Correlation Coefficient

$$maximal\ correlation\ coefficient = \sqrt{\text{second largest eigenvalue of } Q}$$

$$Q = \sum_{k=1}^{N_g} \frac{P(i,k)P(j,k)}{p_x(i)p_y(k)}$$

22. Maximum Probability

$$maximum\ probability = \max\{P(i,j)\}$$

23. Sum average (SA)

$$sum\ average\ (SA) = \sum_{i=2}^{2N_g} \left[iP_{x+y}(i)\right]$$

24. Sum entropy

$$sum\ entropy = -\sum_{i=2}^{2N_g} P_{x+y}(i) \log_2\left[P_{x+y}(i)\right]$$

25. Sum variance

$$sum\ variance = \sum_{i=2}^{2N_g} (i - SA)^2 P_{x+y}(i)$$

26. Variance (sum of squares)

$$variance = \sum_{i=1}^{N_g}\sum_{j=1}^{N_g} (i-\mu)^2 P(i,j)$$

Grey-Level Run-Length matrix based features

Grey-level run-length matrix (GLRLM) based features, as described by Galloway et al. (8). Run length metrics quantify grey level runs in an image. A grey level run is defined as the length in number of pixels, of consecutive pixels that have the same grey level value. In a grey level run length matrix $p(i,j|\theta)$, the (i,j)th element describes the number of times j a grey level i appears consecutively in the direction specified by θ. Let:

$p(i,j)$ be the (i,j)th entry in the given run-length matrix p, generalized for any direction θ,

N_g the number of discrete intensity values in the image,

N_r the maximum run length,

N_s the total number of runs, where $N_s = \sum_{i=1}^{N_g}\sum_{j=1}^{N_r} p(i,j)$,

p_r the sum distribution of the number of runs with run length j, where $p_r(j) = \sum_{i=1}^{N_g} p(i,j)$,

p_g the sum distribution of the number of runs with grey level i, where $p_g(i) = \sum_{j=1}^{N_r} p(i,j)$,

N_p the number of voxels in the image, where $N_p = \sum_{j=1}^{N_r} jp_r$,

$p_n(i,j)$ the normalized run-length matrix, where $p_n(i,j) = \frac{p(i,j)}{N_s}$,

μ_r the mean run length, where $\mu_r = \sum_{i=1}^{N_g} \sum_{j=1}^{N_r} j p_n(i,j)$,

μ_g the mean grey level, where $\mu_g = \sum_{i=1}^{N_g} \sum_{j=1}^{N_r} i p_n(i,j)$.

1. Short Run Emphasis (SRE)

$$SRE = \frac{1}{N_s} \sum_{j=1}^{N_r} \frac{p_r}{j^2}$$

2. Long Run Emphasis (LRE)

$$LRE = \frac{1}{N_s} \sum_{j=1}^{N_r} j^2 p_r$$

3. Grey Level Non-Uniformity (GLN)

$$GLN = \frac{1}{N_s} \sum_{i=1}^{N_g} p_g^2$$

4. Grey Level Non-Uniformity Normalized (GLNN)

$$GLNN = \frac{1}{N_s^2} \sum_{i=1}^{N_g} p_g^2$$

5. Run Length Non-Uniformity (RLN)

$$RLN = \frac{1}{N_s} \sum_{j=1}^{N_r} p_r^2$$

6. Run Length Non-Uniformity Normalized (RLNN)

$$RLNN = \frac{1}{N_s^2} \sum_{j=1}^{N_r} p_r^2$$

7. Run Percentage (RP)

$$RP = \frac{N_s}{N_p}$$

8. Low Grey Level Run Emphasis (LGRE)

$$LGRE = \frac{1}{N_s} \sum_{i=1}^{N_g} \frac{p_g}{i^2}$$

9. High Grey Level Run Emphasis (HGRE)

$$HGRE = \frac{1}{N_s} \sum_{i=1}^{N_g} i^2 p_g$$

10. Short Run Low Grey Level Emphasis (SRLGE)

$$SRLGE = \frac{1}{N_s}\sum_{i=1}^{N_g}\sum_{j=1}^{N_r}\frac{p(i,j)}{i^2 j^2}$$

11. Short Run High Grey Level Emphasis (SRHGE)

$$SRHGE = \frac{1}{N_s}\sum_{i=1}^{N_g}\sum_{j=1}^{N_r}\frac{p(i,j)i^2}{j^2}$$

12. Long Run Low Grey Level Emphasis (LRLGE)

$$LRLGE = \frac{1}{N_s}\sum_{i=1}^{N_g}\sum_{j=1}^{N_r}\frac{p(i,j)j^2}{i^2}$$

13. Long Run High Grey Level Emphasis (LRHGE)

$$LRHGE = \frac{1}{N_s}\sum_{i=1}^{N_g}\sum_{j=1}^{N_r}p(i,j)i^2 j^2$$

14. Grey level variance (GLV)

$$GLV = \sum_{i=1}^{N_g}\sum_{j=1}^{N_r}(i-\mu_g)^2 p_n(i,j)$$

15. Run length variance (RLV)

$$RLV = \sum_{i=1}^{N_g}\sum_{j=1}^{N_r}(j-\mu_r)^2 p_n(i,j)$$

16. Run entropy (RE) (9)

$$RE = -\sum_{i=1}^{N_g}\sum_{j=1}^{N_r}p_n(i,j|\theta)\log_2[p_n(i,j)]$$

Grey-Level size-zone matrix based features

Grey-level size-zone matrix (GLSZM) based features, as described by Thibault et al. (10, 11). A grey level size-zone matrix describes the amount of homogeneous connected areas within the volume, of a certain size and intensity. The (i,j)th entry of the GLSZM $p(i,j)$ is the number of connected areas of grey level (i.e. intensity value) i and size j. GLSZM features therefore describe homogeneous areas within the tumor volume, describing tumor heterogeneity at a regional scale (12). Let:
$p(i,j)$ be the (i,j)th entry in the given GLSZM p,
N_g the number of discrete intensity values in the image,
N_z the size of the largest, homogeneous region in the volume of interest,
N_s the total number of homogeneous regions (zones), where $N_s = \sum_{i=1}^{N_g}\sum_{j=1}^{N_z}p(i,j)$,

p_z the sum distribution of the number of zones with size j, where $p_z(j) = \sum_{i=1}^{N_g} p(i,j)$,
p_g the sum distribution of the number of zones with grey level i, where $p_g(i) = \sum_{j=1}^{N_z} p(i,j)$,
N_p the number of voxels in the image, where $N_p = \sum_{j=1}^{N_z} jp_z$,
$p_n(i,j)$ the normalized size-zone matrix, where $p_n(i,j) = \frac{p(i,j)}{N_s}$,
μ_z the mean zone size, where $\mu_z = \sum_{i=1}^{N_g} \sum_{j=1}^{N_z} jp_n(i,j|\theta)$,
μ_g the mean grey level, where $\mu_g = \sum_{i=1}^{N_g} \sum_{j=1}^{N_z} ip_n(i,j|\theta)$.

1. Small area Emphasis (SAE)

$$SAE = \frac{1}{N_s} \sum_{j=1}^{N_z} \frac{p_z}{j^2}$$

2. Large area Emphasis (LAE)

$$LAE = \frac{1}{N_s} \sum_{j=1}^{N_z} j^2 p_z$$

3. Intensity Non-Uniformity (IN)

$$IN = \frac{1}{N_s} \sum_{i=1}^{N_g} p_g^2$$

4. Intensity Non-Uniformity Normalized (INN)

$$INN = \frac{1}{N_s^2} \sum_{i=1}^{N_g} p_g^2$$

5. Size-zone Non-Uniformity (SZN)

$$SZN = \frac{1}{N_s} \sum_{j=1}^{N_z} p_z^2$$

6. Size-zone Non-Uniformity Normalized (SZNN)

$$SZNN = \frac{1}{N_s^2} \sum_{j=1}^{N_z} p_z^2$$

7. Zone Percentage (ZP)

$$ZP = \frac{N_s}{N_p}$$

8. Low intensity Emphasis (LIE)

$$LIE = \frac{1}{N_s} \sum_{i=1}^{N_g} \frac{p_g}{i^2}$$

9. High intensity Emphasis (HIE)

$$HIE = \frac{1}{N_s}\sum_{i=1}^{N_g} i^2 p_g$$

10. Low intensity small area Emphasis (LISAE)

$$LISAE = \frac{1}{N_s}\sum_{i=1}^{N_g}\sum_{j=1}^{N_z}\frac{p(i,j)}{i^2 j^2}$$

11. High intensity small area Emphasis (HISAE)

$$HISAE = \frac{1}{N_s}\sum_{i=1}^{N_g}\sum_{j=1}^{N_z}\frac{p(i,j)i^2}{j^2}$$

12. Low intensity large area Emphasis (LILAE)

$$LILAE = \frac{1}{N_s}\sum_{i=1}^{N_g}\sum_{j=1}^{N_z}\frac{p(i,j)j^2}{i^2}$$

13. High intensity large area Emphasis (HILAE)

$$HILAE = \frac{1}{N_s}\sum_{i=1}^{N_g}\sum_{j=1}^{N_z} p(i,j)i^2 j^2$$

14. Intensity variance (IV)

$$IV = \sum_{i=1}^{N_g}\sum_{j=1}^{N_z}(i-\mu_g)^2 p_n(i,j)$$

15. Size-zone variance (SZV)

$$SZV = \sum_{i=1}^{N_g}\sum_{j=1}^{N_z}(j-\mu_z)^2 p_n(i,j)$$

16. Zone entropy (ZE)

$$ZE = \sum_{i=1}^{N_g}\sum_{j=1}^{N_z} p_n(i,j)\log_2[p_n(i,j)]$$

Grey-Level distance-zone matrix based features

Grey-level distance-zone matrix (GLDZM) based features, as described by Thibault et al. (13). A grey level distance-zone matrix describes the amount of homogeneous connected areas within the volume, of a certain intensity and distance to the shape border. The shape border is defined by 6-connectedness in 3D (i.e. a voxel is on the border, if at least one face is exposed). In contrast to the original definition by Thibault et al. (13), the minimum distance to the border is 1, instead of 0 (i.e.

voxels on the border have a distance of 1), to allow for correct feature calculations. The (i,j)th entry of the GLDZM $p(i,j)$ is the number of connected areas of grey level (i.e. intensity value) i and minimum distance j to the shape border. GLSZM features therefore describe the radial distribution of homogeneous areas within the tumor volume. Let:
$p(i,j)$ be the (i,j)th entry in the given GLDZM p,
N_g the number of discrete intensity values in the image,
N_d the largest distance of a homogeneous region in the volume of interest to the shape border,
N_s the total number of homogeneous regions (zones), where $N_s = \sum_{i=1}^{N_g} \sum_{j=1}^{N_d} p(i,j)$,
p_d the sum distribution of the number of zones with distance j, where $p_z(j) = \sum_{i=1}^{N_g} p(i,j)$,
p_g the sum distribution of the number of zones with grey level i, where $p_g(i) = \sum_{j=1}^{N_z} p(i,j)$,
N_p the number of voxels in the image, where $N_p = \sum_{j=1}^{N_d} jp_d$,
$p_n(i,j)$ the normalized size-zone matrix, where $p_n(i,j) = \frac{p(i,j)}{N_s}$,
μ_d the mean distance, where $\mu_d = \sum_{i=1}^{N_g} \sum_{j=1}^{N_d} jp_n(i,j|\theta)$,
μ_g the mean grey level, where $\mu_g = \sum_{i=1}^{N_g} \sum_{j=1}^{N_d} ip_n(i,j|\theta)$.

1. Small distance Emphasis (SDE)

$$SDE = \frac{1}{N_s} \sum_{j=1}^{N_d} \frac{p_d}{j^2}$$

2. Large distance Emphasis (LDE)

$$LDE = \frac{1}{N_s} \sum_{j=1}^{N_d} j^2 p_d$$

3. Intensity Non-Uniformity (IN)

$$IN = \frac{1}{N_s} \sum_{i=1}^{N_g} p_g^2$$

4. Intensity Non-Uniformity Normalized (INN)

$$INN = \frac{1}{N_s^2} \sum_{i=1}^{N_g} p_g^2$$

5. Distance-zone Non-Uniformity (DZN)

$$DZN = \frac{1}{N_s} \sum_{j=1}^{N_d} p_d^2$$

6. Distance-zone Non-Uniformity Normalized (DZNN)

$$DZNN = \frac{1}{N_s^2} \sum_{j=1}^{N_d} p_d^2$$

7. Zone Percentage (ZP)

$$ZP = \frac{N_s}{N_p}$$

8. Low intensity Emphasis (LIE)

$$LIE = \frac{1}{N_s} \sum_{i=1}^{N_g} \frac{p_g}{i^2}$$

9. High intensity Emphasis (HIE)

$$HIE = \frac{1}{N_s} \sum_{i=1}^{N_g} i^2 p_g$$

10. Low intensity small distance Emphasis (LISDE)

$$LISDE = \frac{1}{N_s} \sum_{i=1}^{N_g} \sum_{j=1}^{N_d} \frac{p(i,j)}{i^2 j^2}$$

11. High intensity small distance Emphasis (HISDE)

$$HISDE = \frac{1}{N_s} \sum_{i=1}^{N_g} \sum_{j=1}^{N_d} \frac{p(i,j) i^2}{j^2}$$

12. Low intensity large distance Emphasis (LILDE)

$$LILDE = \frac{1}{N_s} \sum_{i=1}^{N_g} \sum_{j=1}^{N_d} \frac{p(i,j) j^2}{i^2}$$

13. High intensity large distance Emphasis (HILDE)

$$HILDE = \frac{1}{N_s} \sum_{i=1}^{N_g} \sum_{j=1}^{N_d} p(i,j) i^2 j^2$$

14. Intensity variance (IV)

$$IV = \sum_{i=1}^{N_g} \sum_{j=1}^{N_d} (i - \mu_g)^2 p_n(i,j)$$

15. Distance-zone variance (DZV)

$$SZV = \sum_{i=1}^{N_g} \sum_{j=1}^{N_d} (j - \mu_d)^2 p_n(i,j)$$

16. Distance-zone entropy (DZE)

$$DZE = \sum_{i=1}^{N_g} \sum_{j=1}^{N_d} p_n(i,j) \log_2 [p_n(i,j)]$$

Neighborhood Grey tone difference matrix based features

Neighborhood Grey tone difference matrix (NGTDM) based features, as described by Amadasun and King (14). The ith entry of the NGTDM $s(i|d)$ is the sum of grey level differences of voxels with intensity i and the average intensity A_i of their neighboring voxels within a distance d. In contrast to the original paper, a complete neighborhood is not required and A_i is determined over the valid voxels. Let:

n_i be the number of voxels with grey level i,

$N_v = \sum n_i$, the total number of voxels (defined as n^2 by Amadasun and King (14)),

$s(i) = \begin{cases} \sum_{n_i} |i - A_i| & \text{for } n_i > 0 \\ 0 & \text{otherwise} \end{cases}$, generalized for any distance d,

N_g be the maximum discrete intensity level in the image,

$p(i) = \frac{n_i}{N_v}$, the probability of grey level i,

N_p, the total number of grey levels present in the image.

1. Coarseness

$$coarseness = \frac{1}{\varepsilon + \sum_{i=i}^{N_g} p(i)s(i)}$$

Where ε is a small number to prevent coarseness becoming infinite.

2. Contrast

$$contrast = \left(\frac{1}{N_p(1 - N_p)} \sum_{i=1}^{N_g} \sum_{j=1}^{N_g} p(i)p(j)(i - j)^2 \right) \left(\frac{1}{N_v} \sum_{i=i}^{N_g} s(i) \right)$$

3. Busyness

$$busyness = \frac{\sum_{i=i}^{N_g} p(i)s(i)}{\sum_{i=i}^{N_g} \sum_{j=i}^{N_g} |ip(i) - jp(j)|}, \qquad p(i) \neq 0,\ p(j) \neq 0$$

4. Complexity

$$complexity = \frac{1}{N_v} \sum_{i=i}^{N_g} \sum_{j=i}^{N_g} |i - j| \frac{p(i)s(i) + p(j)s(j)}{p(i) + p(j)}, \qquad p(i) \neq 0,\ p(j) \neq 0$$

5. Strength

$$strength = \frac{\sum_{i=i}^{N_g} \sum_{j=i}^{N_g} [p(i) + p(j)](i - j)^2}{\varepsilon + \sum_{i=i}^{N_g} s(i)}, \qquad p(i) \neq 0,\ p(j) \neq 0$$

Neighboring Grey level dependence matrix based features

Neighboring Grey level dependence matrix (NGLDM) based features, as described by Sun and Wee (15). NGLDM features are invariant under spatial rotation. The (i, j)th entry of the NGLDM $p(i, j|d, a)$ describes the number of neighborhoods with center voxel grey level (i.e. intensity value) i and

dependence (i.e. number of dependent voxels) $k = j - 1$. A neighborhood are all voxels within a distance d from the center voxel. The center voxel and a neighboring voxel are dependent if their absolute grey value difference $\leq a$, the dependency coarseness parameter. The features originally specified by Sun and Wee are analogous to the GLRLM and GLSZM features, and the feature set is extended accordingly. Let:

$p(i,j)$ be the (i,j)th entry in the given NGLDM p, generalized for any d and a,

N_g the number of discrete intensity values in the image,

N_d the maximum dependence value,

N_s the total number of neighborhoods, where $N_s = \sum_{i=1}^{N_g} \sum_{j=1}^{N_z} p(i,j)$,

p_d the sum distribution of the number of neighborhoods with dependence $j = k + 1$, where $p_d(j) = \sum_{i=1}^{N_g} p(i,j)$,

p_g the sum distribution of the number of neighborhoods with center voxel grey level i, where $p_g(i) = \sum_{j=1}^{N_d} p(i,j)$,

$p_n(i,j)$ the normalized NGLDM, where $p_n(i,j) = \frac{p(i,j)}{N_s}$,

μ_d the mean dependence, where $\mu_r = \sum_{i=1}^{N_g} \sum_{j=1}^{N_z} j p_n(i,j|\theta)$,

μ_g the mean grey level, where $\mu_g = \sum_{i=1}^{N_g} \sum_{j=1}^{N_z} i p_n(i,j|\theta)$.

Note: By definition, the number of voxels in the image (N_p) equals the total number of neighborhoods (N_s), since in our implementation every voxel is considered to have a neighborhood. Feature "dependence percentage" $\left(\frac{N_s}{N_p}\right)$, which is the equivalent to run-length feature "run percentage" (RP; 7), is therefore omitted, because it will always evaluate to 1.

1. Small Dependence Emphasis (SDE)

$$SDE = \frac{1}{N_s} \sum_{j=1}^{N_d} \frac{p_d}{j^2}$$

This feature is also called Small Number Emphasis (15).

2. Large Dependence Emphasis (LDE)

$$LDE = \frac{1}{N_s} \sum_{j=1}^{N_d} j^2 p_d$$

This feature is also called Large Number Emphasis (15).

3. Grey-level Non-Uniformity (GLN)

$$GLN = \frac{1}{N_s} \sum_{i=1}^{N_g} p_g^2$$

4. Grey-level Non-Uniformity Normalized (GLNN)

$$GLNN = \frac{1}{N_s^2} \sum_{i=1}^{N_g} p_g^2$$

5. Dependence Non-Uniformity (DN)

$$DN = \frac{1}{N_s}\sum_{j=1}^{N_d} p_z^2$$

This feature is also called Number Nonuniformity (15).

6. Dependence Non-Uniformity Normalized (DNN)

$$DNN = \frac{1}{N_s^2}\sum_{j=1}^{N_z} p_z^2$$

7. Low Grey-level Emphasis (LGE)

$$LGE = \frac{1}{N_s}\sum_{i=1}^{N_g} \frac{p_g}{i^2}$$

8. High Grey-level Emphasis (HGE)

$$HGE = \frac{1}{N_s}\sum_{i=1}^{N_g} i^2 p_g$$

9. Low Grey-level small Dependence Emphasis (LGSDE)

$$LGSDE = \frac{1}{N_s}\sum_{i=1}^{N_g}\sum_{j=1}^{N_d} \frac{p(i,j)}{i^2 j^2}$$

10. High Grey-level small Dependence Emphasis (HGSDE)

$$HGSDE = \frac{1}{N_s}\sum_{i=1}^{N_g}\sum_{j=1}^{N_d} \frac{p(i,j)i^2}{j^2}$$

11. Low Grey-level large Dependence Emphasis (LGLDE)

$$LGLDE = \frac{1}{N_s}\sum_{i=1}^{N_g}\sum_{j=1}^{N_d} \frac{p(i,j)j^2}{i^2}$$

12. High Grey-level large Dependence Emphasis (HGLDE)

$$HGLDE = \frac{1}{N_s}\sum_{i=1}^{N_g}\sum_{j=1}^{N_d} p(i,j)i^2 j^2$$

13. Grey-level variance (GLV)

$$GLV = \sum_{i=1}^{N_g}\sum_{j=1}^{N_d} (i-\mu_g)^2 p_n(i,j)$$

14. Dependence variance (DV)

$$DV = \sum_{i=1}^{N_g}\sum_{j=1}^{N_d}(j-\mu_d)^2 p_n(i,j)$$

15. Dependence entropy (DE), also called Entropy (15)

$$DE = \sum_{i=1}^{N_g}\sum_{j=1}^{N_d} p_n(i,j)\log_2[p_n(i,j)]$$

Note: the definition of entropy by Sun and Wee (15) uses the dependence counts (p) instead of the dependence propabilities (p_n).

16. Second moment (SM) (15)

$$SM = \frac{\sum_{i=1}^{N_g}\sum_{j=1}^{N_d} p(i,j)^2}{N_s}$$

Note: for this feature, defined by Sun and Wee (15), there is no grey-level run-length equivalent

Wavelet features

Wavelet transform effectively decouples textural information by decomposing the original image, in a similar manner as Fourier analysis, in low– and high-frequencies. In this study a discrete, one-level and undecimated three dimensional wavelet transform was applied to each CT and CBCT image, which decomposes the original image X into 8 decompositions. Consider L and H to be a low-pass (i.e. a scaling) and, respectively, a high-pass (i.e. a wavelet) function, and the wavelet decompositions of X to be labeled as X_{LLL}, X_{LLH}, X_{LHL}, X_{LHH}, X_{HLL}, X_{HLH}, X_{HHL} and X_{HHH}. For example, X_{LLH} is then interpreted as the high-pass sub band, resulting from directional filtering of X with a low-pass filter along x-direction, a low pass filter along y-direction and a high-pass filter along z-direction and is constructed as:

$$X_{LLH}(i,j,k) = \sum_{p=1}^{N_L}\sum_{q=1}^{N_L}\sum_{r=1}^{N_H} L(p)L(q)H(r)X(i+p,j+q,k+r)$$

Where N_L is the length of filter L and N_H is the length of filter H. The other decompositions are constructed in a similar manner, applying their respective ordering of low or high-pass filtering in x, y and z-direction. Wavelet decomposition of the image X is schematically depicted in Figure 7. Since the applied wavelet decomposition is undecimated, the size of each decomposition is equal to the original image and each decomposition is shift invariant. Because of these properties, the original tumor delineation of the gross tumor volume (GTV) can be applied directly to the decompositions after wavelet transform. In this study "Coiflet 1" wavelet was applied. For each decomposition we computed the first order gray level statistics and the textural features (GLCM, GLRLM, GLSZM).

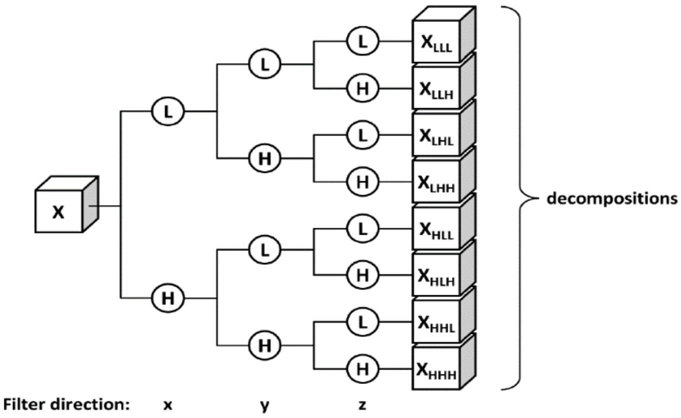

Figure 8: Schematic of the undecimated three dimensional wavelet transform applied to each CT/CBCT image. The original image X is decomposed into 8 decompositions, by directional low-pass (i.e. a scaling) and high-pass (i.e. a wavelet) filtering: X_{LLL}, X_{LLH}, X_{LHL}, X_{LHH}, X_{HLL}, X_{HLH}, X_{HHL} and X_{HHH}.
